# Involvement of PKA/DARPP-32/PP1α and β- arrestin/Akt/GSK-3β Signaling in Cadmium-Induced DA-D2 Receptor-Mediated Motor Dysfunctions: Protective Role of Quercetin

**DOI:** 10.1038/s41598-018-20342-z

**Published:** 2018-02-06

**Authors:** Richa Gupta, Rajendra K. Shukla, Ankita Pandey, Tanuj Sharma, Yogesh K. Dhuriya, Pranay Srivastava, Manjul P. Singh, Mohammad Imran Siddiqi, Aditya B. Pant, Vinay K. Khanna

**Affiliations:** 1 Developmental Toxicology Laboratory, Systems Toxicology and Health Risk Assessment Group, CSIR-Indian Institute of Toxicology Research, Vishvigyan Bhawan, 31 Mahatma Gandhi Marg, Lucknow, 226 001 Uttar Pradesh India; 2 School of Pharmacy, Babu Banarsi Das University, Faizabad Road, Lucknow, 226 028 Uttar Pradesh India; 3 Molecular and Structural Biology, CSIR-Central Drug Research Institute, Sitapur Road, Sector 10, Jankipuram Extension, Lucknow, 226 031 Uttar Pradesh India

## Abstract

Given increasing risk of cadmium-induced neurotoxicity, the study was conducted to delineate the molecular mechanisms associated with cadmium-induced motor dysfunctions and identify targets that govern dopaminergic signaling in the brain involving *in vivo*, *in vitro*, and *in silico* approaches. Selective decrease in dopamine (DA)-D2 receptors on cadmium exposure was evident which affected the post-synaptic PKA/DARPP-32/PP1α and β-arrestin/Akt/GSK-3β signaling concurrently in rat corpus striatum and PC12 cells. Pharmacological inhibition of PKA and Akt *in vitro* demonstrates that both pathways are independently modulated by DA-D2 receptors and associated with cadmium-induced motor deficits. Ultrastructural changes in the corpus striatum demonstrated neuronal degeneration and loss of synapse on cadmium exposure. Further, molecular docking provided interesting evidence that decrease in DA-D2 receptors may be due to direct binding of cadmium at the competitive site of dopamine on DA-D2 receptors. Treatment with quercetin resulted in the alleviation of cadmium-induced behavioral and neurochemical alterations. This is the first report demonstrating that cadmium-induced motor deficits are associated with alteration in postsynaptic dopaminergic signaling due to a decrease in DA-D2 receptors in the corpus striatum. The results further demonstrate that quercetin has the potential to alleviate cadmium-induced dopaminergic dysfunctions.

## Introduction

Cadmium, a heavy metal with wide occurrence in nature has extensive industrial and anthropogenic uses because of its non-corrosive nature and thus enhances the risk of human exposure. Presence of high cadmium levels in cigarette smoke and drinking water are potential sources of non-occupational exposure^1^. Exposure to cadmium could also occur through the ambient air in urban areas close to industrial settings^2^. As cadmium has a high rate of transfer from soil to plants, vegetables, fruits and cereals have been found to be contaminated^3–5^. The general population, therefore, is exposed to cadmium through food chain while consuming dietary products. The biological half-life of cadmium in the human body is around 15–20 years. Due to poor elimination, it is cumulative in nature and gets distributed in body organs^1^. Although cadmium has been found to be carcinogenic, disruption in the functioning of lungs, liver, and kidneys has been reported extensively in cadmium-exposed individuals^2,6^. Increasing incidences of neurological and psychiatric disturbances associated with cognitive deficits on cadmium exposure in recent years is a cause for concern and reflect the vulnerability of brain^7–9^. Risk of Alzheimer disease has been associated with cadmium exposure as high cadmium levels in plasma, brain and liver were detected in Alzheimer’s patients^9–12^.

Role of dopaminergic neurotransmission in the regulation of motor and reward system and in the etiology of neurodegenerative disorders has been demonstrated extensively^13^. Of the two classes of dopamine (DA) receptors, DA-D1 type receptors include DA-D1 and DA-D5 subtypes and increase cAMP levels on stimulation with Gs units. In contrast, the DA-D2 type receptors include DA-D2, DA-D3, and DA-D4 subtypes and decrease cAMP levels by interacting with Gi units. Interestingly, the DA-D2 receptors mediate behavior either by cAMP-dependent PKA phosphorylation leading to altered DARPP-32/PP1α signaling or cAMP independent Akt/GSK-3β signaling pathways^14,15^. Emerging literature reveals that cadmium causes long-lasting neurological abnormalities and impairs motor functions. In a cross-sectional epidemiological study, cadmium levels in urine were inversely associated with visuomotor functions, peripheral neuropathy and disturbed equilibrium^7^. Acute exposure to cadmium has been found to cause Parkinson’s disease^16^. Despite considerable investigations on cadmium neurotoxicity and moreover enhanced risk to develop Parkinson’s disease, the exact mechanism by which it causes brain dopaminergic alterations and affects dopamine-dependent behavior is not well understood.

Given increasing risk of cadmium-induced neurotoxicity, a number of experimental studies have been carried out involving pharmacological and natural extracts to assess their protective potential. Among flavonoids, quercetin, a class of flavonol is widely present in vegetables, fruits, tea and many other foods and medicinal plants^17–19^. The high antioxidant capacity of quercetin has made it popular over other antioxidants including vitamin C, vitamin E, and β-carotene. Potential of quercetin to chelate transition metals has been found to protect iron-induced Fenton reaction^20,21^. Although anti-carcinogenic, anti-inflammatory and vasodilating effects of quercetin are well documented, it has been found effective in the management of nervous system disorders including Parkinson’s and Huntington’s diseases^22,23^. The present study has been carried out to identify the molecular targets associated with cadmium-induced brain dopaminergic alterations involving *in vivo, in vitro*, and *in silico* approaches. The study also aims to understand the functional deficits associated with cadmium-induced dopaminergic dysfunctions. Further, the potential of quercetin to ameliorate cadmium-induced brain dopaminergic alterations has also been assessed.

## Results

### *In vivo* Studies

#### Effect of cadmium, quercetin and their simultaneous treatment on the expression of key proteins involved in presynaptic dopaminergic signaling

**Figure 1:**
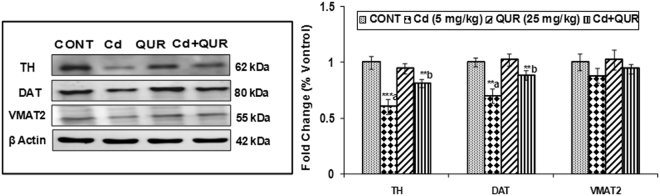
Effect of cadmium, quercetin and their simultaneous treatment on the expression of tyrosine hydroxylase, dopamine transporter and vesicular monoamine transporter proteins in the corpus striatum of rats. Quantitative analysis revealed that simultaneous treatment with cadmium & quercetin protected cadmium-induced decrease in the expression of tyrosine hydroxylase (TH), dopamine transporter (DAT) and vesicular monoamine transporter proteins (VMAT-2). Relative protein levels were quantified using Alpha Ease FC Stand Alone V.4.0 software after normalized with β-actin. The cropped gels are displayed for the clear representation. Values are expressed as mean ± SEM of three rats in each group; Significantly differs (**p < 0.01; ***p < 0.001); a-compared to control, b-compared to cadmium exposed group.

Exposure of rats to cadmium for 28 days resulted in the decrease of tyrosine hydroxylase (TH) levels, a rate-limiting enzyme in dopamine synthesis in the corpus striatum (F_(3,8)_ = 11.12, 1.62 fold, p < 0.01) as compared to controls. A decrease in the levels of dopamine transporter (DAT, F_(3,8)_ = 8.176, 1.69 fold, p < 0.01) and vesicle monoamine transporter2 (VMAT2, F_(3,8)_ = 5.183, 0.50 fold, p > 0.05) proteins was also distinct in rats on cadmium exposure. Treatment with quercetin in cadmium-exposed rats caused the upregulation in TH (F_(3,8)_ = 11.12, 1.35 fold, p < 0.05), DAT (F_(3,8)_ = 8.176, 1.27 fold, p < 0.05) and VMAT2 (F_(3,8)_ = 5.183, 0.39 fold, p > 0.05) as compared to rats exposed to cadmium alone. However, there was no significant change in the levels of any of these proteins in the corpus striatum of rats exposed to quercetin alone as compared to controls (Fig. 1).

#### Transcriptional and translational changes in dopamine receptors

**Figure 2:**
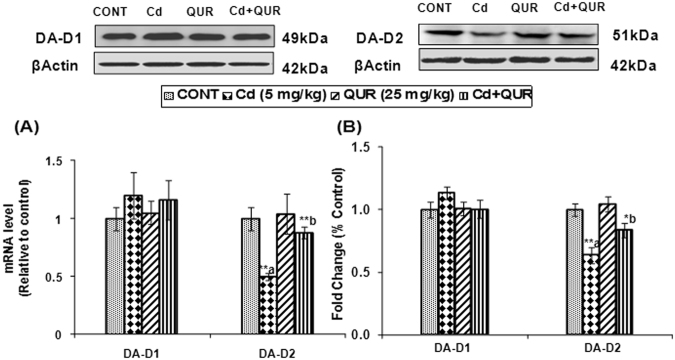
Effect of cadmium, quercetin and their simultaneous treatment on the expression of dopamine receptors in the corpus striatum of rats. Cadmium exposure decreased mRNA (**A**) and protein (**B**) expression of DA-D2 receptors in the corpus striatum while simultaneous treatment with quercetin protected cadmium-induced changes. β-actin was used as a housekeeping gene in qRT-PCR studies and as a loading control in western blotting. The cropped gels are displayed for the clear representation. Values are mean ± SEM of three rats in each group; Significantly differs (*p < 0.05, **p < 0.01); a-compared to control group; b-compared to cadmium exposed group.

As dopamine receptors have a central role in dopaminergic signaling, transcriptional and translational changes in DA-D1 and DA-D2 receptor types in the corpus striatum were assessed on cadmium exposure in rats. No significant change in mRNA expression and protein levels of DA-D1 receptors was observed in the corpus striatum of cadmium-exposed rats. Exposure to cadmium however, resulted in a decrease of mRNA expression (F_(3,8)_ = 7.662, 50%, p < 0.01) and protein levels (F_(3,8)_ = 10.07, 1.63 fold, p < 0.01) of DA-D2 receptors in the corpus striatum as compared to controls (Fig. 2). Interestingly, treatment with quercetin in cadmium-exposed rats caused significant upregulation in mRNA expression (F_(3,8)_ = 7.662, 76%, p < 0.05) and protein level (F_(3,8)_ = 10.07, 1.31 fold, p < 0.05) of DA-D2 receptors as compared to those treated with cadmium alone. No transcriptional and translational changes in DA-D1 and DA-D2 receptor types were observed in the corpus striatum of rats exposed to quercetin alone as compared to controls (Fig. 2).

#### Assessment of DA-D2 receptor binding

**Figure 3:**
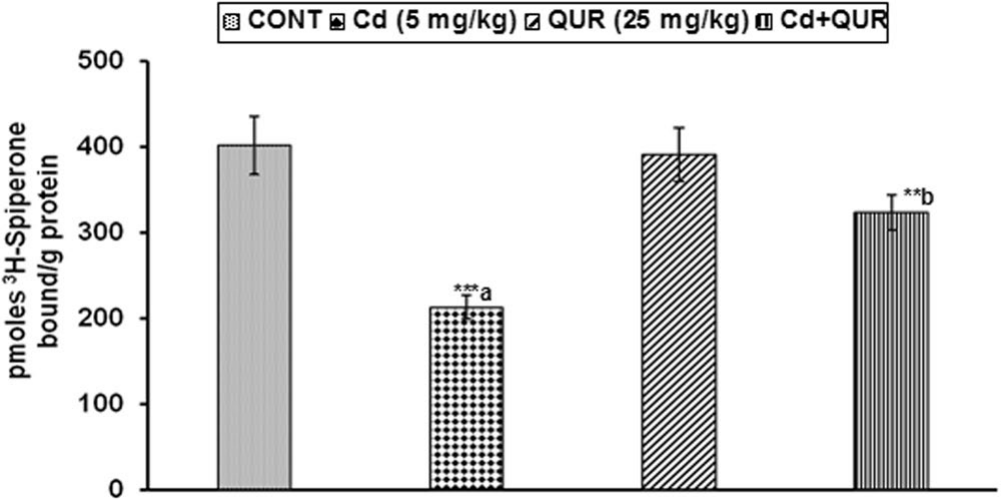
Effect of cadmium, quercetin and their simultaneous treatment on ^3^H-spiperone binding in the corpus striatum of rats. Cadmium exposure decreased the binding of ^3^H-spiperone, known to label DA - D2 receptors while simultaneous treatment with quercetin prevented the decrease. Values are mean ± SEM of five rats in each group; Significantly differs (**p < 0.01, ***p < 0.001); a-compared to control group; b-compared to cadmium exposed group.

To further confirm cadmium-induced changes in the expression of DA-D2 receptors, the effect on the binding of DA-D2 receptors was assessed in the corpus striatum. Statistical analysis revealed that there was a significant decrease (F_(3,16)_ = 11.13, 47%, p < 0.001) in the binding of ^3^H-spiperone to striatal membranes, known to label DA-D2 receptors on exposure of rats to cadmium as compared to controls. Scatchard analysis revealed that decrease in the binding of DA-D2 receptors in the corpus striatum on cadmium exposure was due to alteration in the number of receptor binding sites and no change in the binding affinity (Table 1). Interestingly, treatment with quercetin was found to alleviate cadmium-induced changes in the binding of DA-D2 receptors in the corpus striatum (F_(3,16)_ = 11.13, 51%, p < 0.01) as compared to rats treated with cadmium alone (Fig. 3). Further, no change in the binding of DA-D2 receptors was observed in rats exposed to quercetin alone as compared to controls.

**Table 1:** Scatchard analysis of ^3^H-Spiperone binding to corpus striatal membranes of rats.

Brain Region/Kinetic Parameters	Treatment group			
	CONT	Cd (5 mg/kg)	QUR (25 mg/kg)	Cd + QUR
**Corpus Striatum**				
B_max_	1011 ± 88	696 ± 51^*a^	964 ± 58	913 ± 60^*b^
Kd	1.18 ± 0.24	1.32 ± 0.19	1.23 ± 1.6	1.31 ± 0.19

Values are mean ± SEM of five rats in each group**;** Significantly differs (*p < 0.05); a- compared to control group; b-compared to cadmium exposed group; Kd – Dissociation constant expressed as nM, Bmax – Maximum number of binding sites expressed as pmoles ^3^H-Spiperone bound/g protein.

#### Effect of cadmium, quercetin and their simultaneous treatment on DA-D2 receptor-mediated PKA signaling

**Figure 4:**
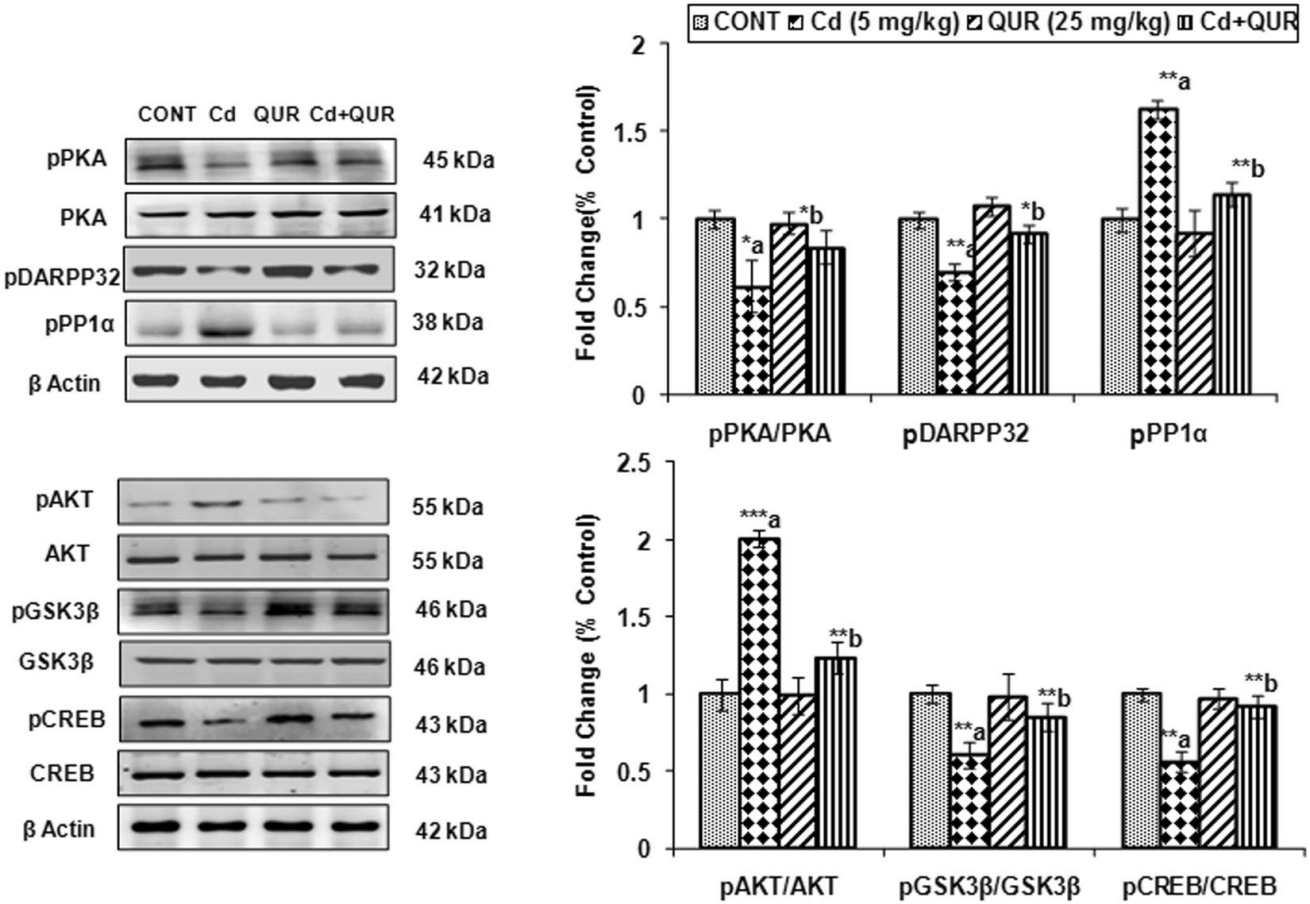
Effect of cadmium, quercetin and their simultaneous treatment on the expression of DA-D2 receptor-mediated targets associated with postsynaptic signaling pathway in the corpus striatum of rats. Quantitative analysis revealed that cadmium exposure down-regulated the expression of PKA, DARPP-32, GSk-3β, CREB and upregulated the expression of Akt, and PP1α while simultaneous treatment with quercetin protected these changes. Relative protein levels were quantified using Alpha Ease FC Stand Alone V.4.0 software after normalization with β-actin. The cropped gels are displayed for the clear representation. Values are mean ± SEM of three rats in each group; Significantly differs (*p < 0.05, **p < 0.01, ***p < 0.001); a-compared to control group; b-compared to cadmium exposed group.

As phosphorylation of DARPP-32 at threonine- 34 by PKA activates the inhibitory function of DARPP-32 over the protein phosphates (PP1α) and affects motor functions, the effect on key targets associated with DA-D2 receptor-mediated downstream signaling was assessed in the corpus striatum. Treatment with cadmium in rats for 28 days decreased the phosphorylation of PKA (F_(3,8)_ = 5.921, 1.63 fold, p < 0.05), DARPP-32 (F_(3,8)_ = 12.38, 1.7 fold, p < 0.01) and CREB (F_(3,8)_ = 8.752, 1.66 fold, p < 0.01) in the corpus striatum as compared to controls. A significant increase in the phosphorylation of PP1α (F_(3,8)_ = 11.96, 1.62 fold, p < 0.01), a negative regulator of DARPP-32 was observed in cadmium exposed rats (Fig. 4). Treatment with quercetin in cadmium exposed rats significantly increased the phosphorylation of PKA (F_(3,8)_ = 5.921, 1.38 fold, p < 0.05), DARPP-32 (F_(3,8)_ = 12.38, 1.3 fold, p < 0.05) and CREB (F_(3,8)_ = 8.752, 1.38 fold, p < 0.01) and decreased the phosphorylation of PP1α (F_(3,8)_ = 11.96, 1.72 fold, p < 0.01) as compared to rats exposed to cadmium alone. Treatment with quercetin in rats had no significant effect on the levels of any of these proteins in the corpus striatum as compared to controls (Fig. 4).

#### Effect of cadmium, quercetin and their simultaneous treatment on cell survival pathway involving β-arrestin-mediated signaling and regulation of Akt by dopamine

As β-arrestin-mediated signaling has an important modulatory role in dopamine-dependent behavior; the involvement of the pathway was assessed on cadmium exposure. An increase in the phosphorylation of Akt (F _(3,8)_ = 17.90, 2.01 fold, p < 0.001) associated with decreased phosphorylation of GSK-3β (F_(3,8)_ = 9.958, 1.62 fold p < 0.01) was evident in the corpus striatum of cadmium-exposed rats as compared to controls. Treatment with quercetin in rats was found to alleviate cadmium-induced changes in the levels of Akt (F_(3,8)_ = 7.662, 1.6 fold, p < 0.01) and GSK-3β (F_(3,8)_ = 9.958, 1.42 fold, p < 0.01) as compared to rats treated with cadmium alone suggesting that quercetin may modulate the cell survival pathway (Fig. 4).

#### Degeneration of striatal neurons

**Figure 5:**
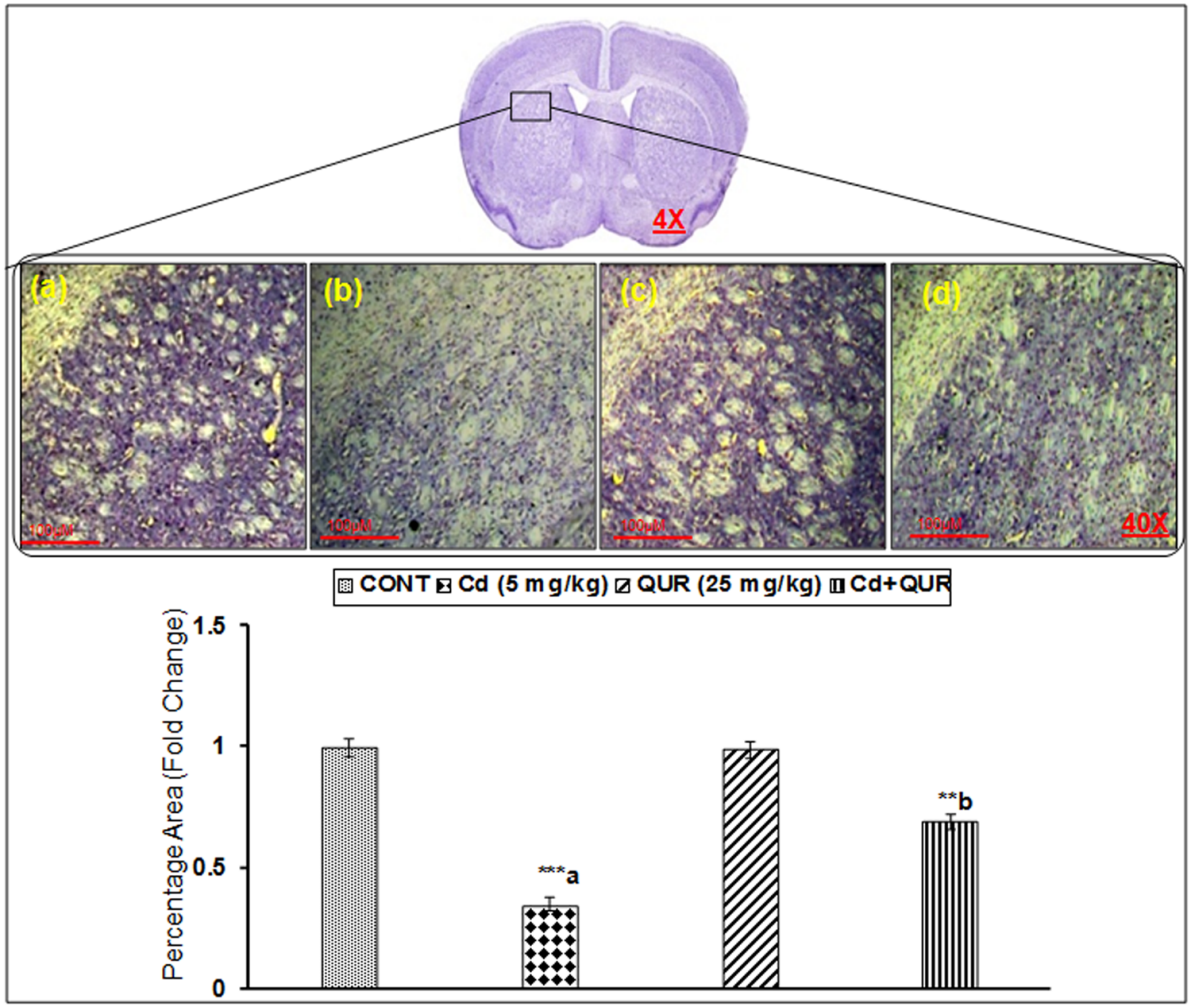
Representative images of Nissl staining of the corpus striatum sections of rat illustrating severe degeneration of neurons of rats exposed to cadmium and quercetin alone or simultaneously for 28 days, (**a**) Control (**b**) Cadmium treated rats (**c**) Quercetin treated rats (**d**) Cd + Qur treated rats. Degeneration of Nissls positive neurons was found in cadmium exposed rats while quercetin protects such degeneration. Values are mean ± SEM of three rats in each group; Significantly differs (**p < 0.01, ***p < 0.001); a-compared to control group; b-compared to cadmium exposed group; Scale bar = 100 μm.

Effect on neuronal degeneration on exposure of rats to cadmium and ameliorative effect of quercetin was assessed in the corpus striatum using cresyl violet staining (Nissl staining). Cadmium exposure resulted in a decrease of neuronal density (F_(3,8)_ = 9.876, 2.01 fold, p < 0.001) as evident by reduced Nissl bodies in the corpus striatum, compared to controls suggesting that cadmium may cause neuronal degeneration. Interestingly, simultaneous exposure with quercetin alleviated cadmium-induced changes in the neuronal density (F_(3,8)_ = 9.876, 1.89 fold, p < 0.001) in the corpus striatum as compared to rats treated with cadmium alone (Fig. 5). Exposure of rats to quercetin alone did not affect the Nissl staining in the corpus striatum.

#### Ultrastructural changes in the corpus striatum

**Figure 6:**
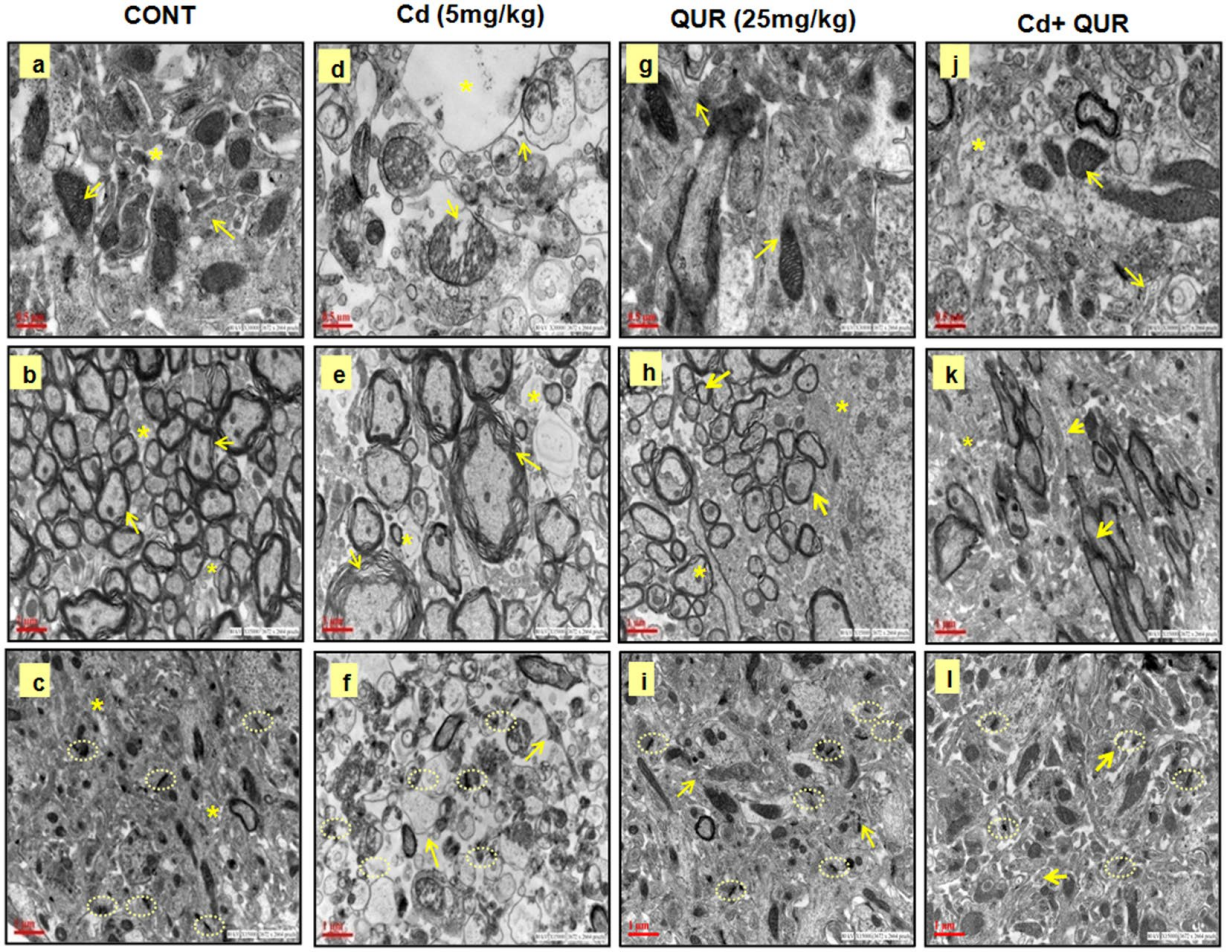
Ultrastructural changes in the corpus striatum of rats. Electron micrographs (30000×) have been presented. (**a–c**) Control group; a- exhibiting the normal anatomy of unmyelinated neurons showing well-developed mitochondria with dense cristae and other organelles; (**b**) well-developed myelin sheath; (**c**) completely developed synapse in neuropil regions. (**d–f**) Cadmium exposed group; (**d**) the loss in cell organelles, vacuole formation in cytoplasm, mitochondria are damaged, swollen mitochondria with loss in membrane cristae (cracked or missing cristae) along with disruption of mitochondrial membrane in unmyelinated axons; (**e**) demyelination of neurons; (**f**) loss of synapse in neuropil regions. (**g–i**) Quercetin exposed group; (**g**) showing normal architecture, with well-developed mitochondria and other cell organelles, (**h**) well developed myelinated neurons; (**i**) well-developed synapse in neuropil region. (**j–l**) Group of rats simultaneously exposed  to cadmium and quercetin, cadmium induced changes were found to be protected on co-exposure with quercetin.

Treatment with cadmium in rats for 28 days caused a marked deterioration in the corpus striatum as visualized by the loss of neurons, disturbed cell matrix, vacuolization associated with damage to the cell organelles including mitochondria. Disruption in the myelin sheath in the corpus striatum was frequently observed on cadmium exposure. The synaptic loss represented by decreased synapse in neuropil region was prominent which could be associated with altered dopaminergic signaling as compared to controls. Simultaneous treatment with quercetin was found to alleviate cadmium-induced ultrastructural changes (Fig. 6). The ultrastructural integrity was found intact in the corpus striatum of rats exposed to quercetin alone.

### Behavioral Studies

#### Spontaneous motor activity

**Figure 7:**
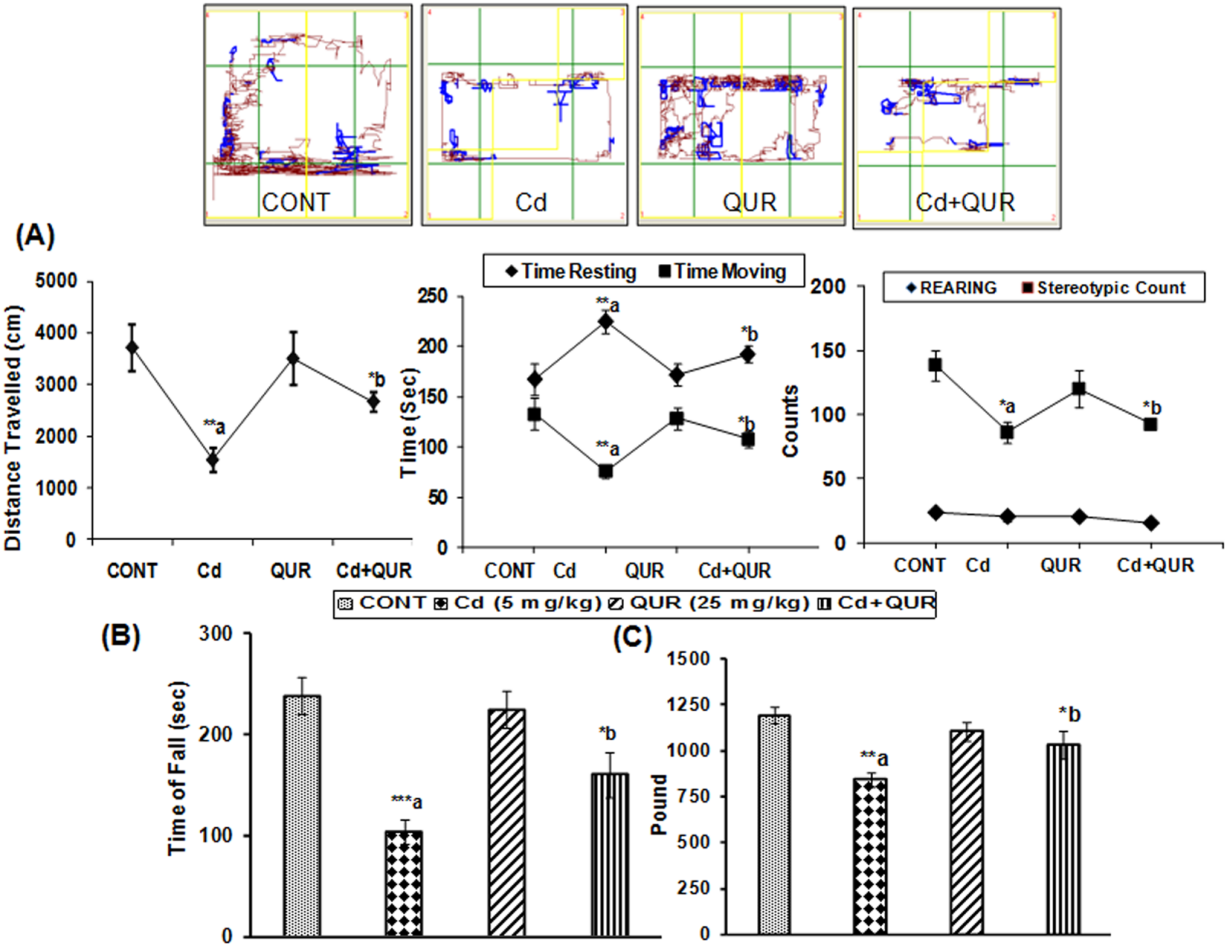
Effect on spontaneous motor activity (**A**), motor coordination (**B**) and muscle strength (**C**) of rats. The decrease in distance traveled, time moving and stereotypic count and increased resting time was observed in cadmium-treated rats while improvement was observed in these rats treated with quercetin simultaneously. Tracking patterns were obtained from automated software ActiMot2 while monitoring the open field activity of rats in different treatment groups. Representative tracking pattern obtained has been shown in each group.Impairment in motor coordination and grip strength was observed in cadmium-exposed rats which also improved on simultaneous treatment with quercetin.Values are mean ± SEM of five rats in each group; Significantly differs (*p < 0.05, **p < 0.01); a-compared to control group; b-compared to cadmium exposed group.

While monitoring the open field activity using ACTIMOT, cadmium treated rats were found to have decreased total distance travelled (F_(3,17)_ = 6.521, 58%, p < 0.001), time moving (F_(3,17)_ = 5.553, 43%, p < 0.05), rearing (F_(3,17)_ = 2.754, 10%, p > 0.05) and stereotypic counts (F_(3,17)_ = 5.651, 38%, p < 0.05) as compared to control rats. Interestingly, these changes were associated with increase in time resting (F_(3,17)_ = 5.655,35%, p < 0.01) in cadmium treated rats. A significant increase in total distance travelled (F_(3,17)_ = 6.521, 73%, p < 0.05), time moving (F_(3,17)_ = 5.553, 42%, p < 0.05), rearing (F_(3,17)_ = 2.754, 25%, p > 0.05) and stereotypic count (F_(3,17)_ = 5.655, 10%, p > 0.05) associated with decrease in time resting (F_(3,17)_ = 5.655,16.8% p < 0.05) was clearly evident in cadmium treated rats simultaneously exposed with quercetin (Fig. 7A). No significant change in any of these parameters was observed in rats treated with quercetin alone as compared to controls.

#### Effect on rotarod performance

Effect on motor coordination was assessed by monitoring the performance using rota-rod. Cadmium-exposed rats fell earlier from the rotating rod and thus evinced impaired motor coordination (F_(3,16)_ = 12.77, 56%, p < 0.001) as compared to controls. Rats simultaneously treated with cadmium and quercetin were found to stay longer on the rotating rod (F_(3,16)_ = 12.77, 55%, p < 0.05) as compared to those treated with cadmium alone. Treatment with quercetin alone had no effect on the time of fall from the rotating rod as compared to control rats (Fig. 7B).

#### Effect on grip strength

A significant impairment (F_(3,16)_ = 7.296, 29%, p < 0.01) in forelimb grip strength was observed in rats exposed to cadmium as compared to rats in the control group. Interestingly, there was a significant improvement (F_(3,16)_ = 7.296, 22.6%, p < 0.05) in forelimb strength of rats simultaneously treated with cadmium and quercetin as compared to those treated with cadmium alone. Further, no significant change in forelimb grip strength was observed in rats exposed to quercetin alone (Fig. 7C).

### *In vitro* Studies

#### Neuronal differentiation of PC12 cells and cytotoxicity studies

**Figure 8:**
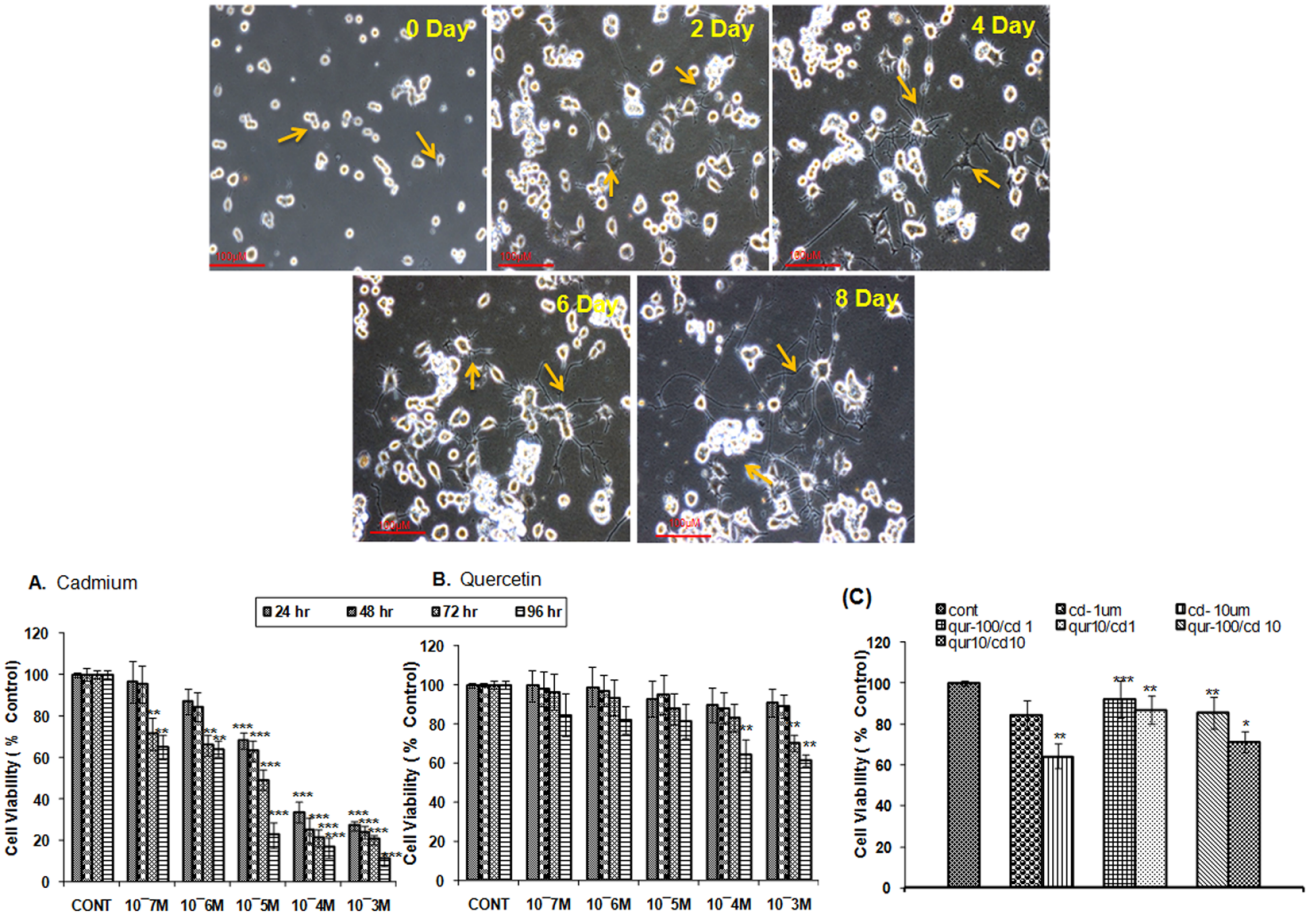
Assessment of non-cytotoxic and cytoprotective doses of cadmium and quercetin. % cell viability in PC12 cells was assessed by MTT assay using different concentrations of cadmium and quercetin at different time intervals (24−96 h). Values are mean ± SEM of three experiments each carried out in triplicate. Significantly differs (**p < 0.01, ***p < 0.001). Scale bar = 100 μm.

PC12 cells were seeded on the poly-L-Lysine coated flasks and differentiated in medium containing NGF (100 ng/ml) for 8 days. These cells were fully differentiated into neurons. Representative images of neuronal differentiation of PC12 cells at 0, 2, 4, 6 and 8 days are presented (Fig. 8). Effect of cadmium and quercetin was assessed at different concentrations (10^−3^ to 10^−7^ M) and at four different time points (24–96 hr) on the viability of differentiated PC12 cells. The non cytotoxic concentration of cadmium and biological safe concentration of quercetin was determined by MTT assay. Simultaneous exposure with quercetin at 100 µM concentration was found to alleviate cytotoxicity significantly in PC12 cells exposed to cadmium both at 1 and 10 µM as revealed by MTT assay (Fig. 8).

#### Effect of cadmium, quercetin and their simultaneous treatment on the expression of key proteins involved in presynaptic dopaminergic signaling

**Figure 9:**
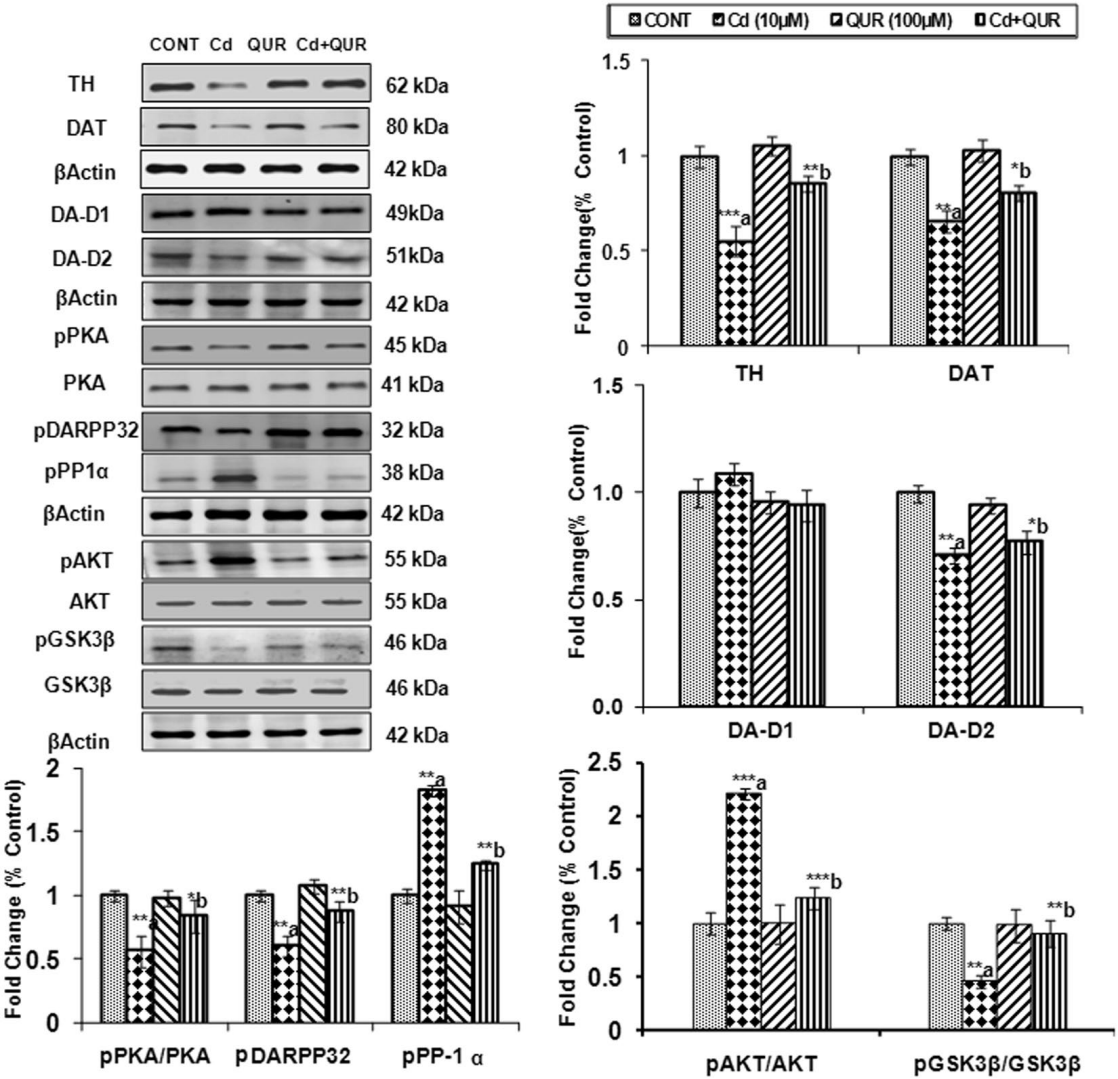
Effect on the expression of dopamine receptors and its targets associated with pre and postsynaptic signaling in differentiated PC12 cells following the exposures of cadmium (10 μM) and quercetin (100 μM). Quantitative analysis revealed that PC12 cells were exposed to Cd (10 µM) and quercetin (100 µM) for 24 hr alters the pre and postsynaptic signaling targets. β-actin was used as an internal control to normalize the data. Relative protein levels were quantified using Alpha Ease FC Stand Alone V.4.0 software after normalization with β-actin. The cropped gels are displayed for the clear representation.The data represent means ± SE of three independent experiments; *p < 0.05; **p < 0.01; a-compared to control group; b-compared to cadmium exposed group; CONT – Control, Cd – Cadmium, QUR – Quercetin.

After establishing the non-cytotoxic and cytoprotective concentration of cadmium and quercetin respectively, ameliorative effect of quercetin on the expression of proteins associated with pre-dopaminergic signaling (TH, DAT) was assessed in PC12 cells. Exposure of differentiated PC12 cells to cadmium (10 µM) for 24 hr resulted in causing a decrease in the expression of TH and DAT as compared to the cells which were not exposed to cadmium (Fig. 9). Treatment with quercetin (100 µM) was found to alleviate cadmium-induced changes in the levels of these proteins associated with pre-dopaminergic signaling.

#### Effect of cadmium, quercetin and their simultaneous treatment on the expression of dopamine receptors

Although there was no change in the expression of DA-D1 receptors on exposure to cadmium (10 µM), decrease in the levels of DA-D2 receptors was clearly evident (Fig. 9). Interestingly, changes were found consistent with *in vivo* study and indicate vulnerability of DA-D2 receptors to cadmium. Treatment with quercetin was found to attenuate cadmium-induced decrease in the levels of DA-D2 receptors and thus exhibits the potential of quercetin in preventing such changes.

#### Effect of cadmium, quercetin and their simultaneous treatment on DA-D2 receptor-mediated postsynaptic signaling

Exposure of PC12 cells to cadmium resulted in a decrease of pPKA, pDARPP-32 and pCREB phosphorylation suggesting that cadmium affects the DA-D2 receptor-mediated down streaming signaling (Fig. 9). It was interesting to note that phosphorylation of pPP1α, a negative regulator of DARPP-32 was increased on treatment with cadmium in PC12 cells indicating that dopamine-dependent signaling is vulnerable to cadmium exposure. Treatment with quercetin was found to attenuate cadmium-induced changes in the levels of these proteins (Fig. 9).

#### Effect of cadmium, quercetin and their simultaneous treatment on β-arrestin-mediated cell signaling pathway

Consistent with *in vivo* findings, exposure to cadmium was found to inhibit DA-D2 receptor-mediated β-arrestin2/Akt/GSK-3β survival pathway which plays an important role in DA-D2 receptor-mediated behavioral functions. Cadmium exposure in PC12 cells resulted in an increase of Akt phosphorylation associated with decreased phosphorylation of GSK-3β. Simultaneous exposure with quercetin was found to protect cadmium-induced changes in the levels of Akt and GSK-3β in PC12 cells as compared to cadmium-treated cells alone (Fig. 9).

#### Studies with pharmacological inhibitors

**Figure 10:**
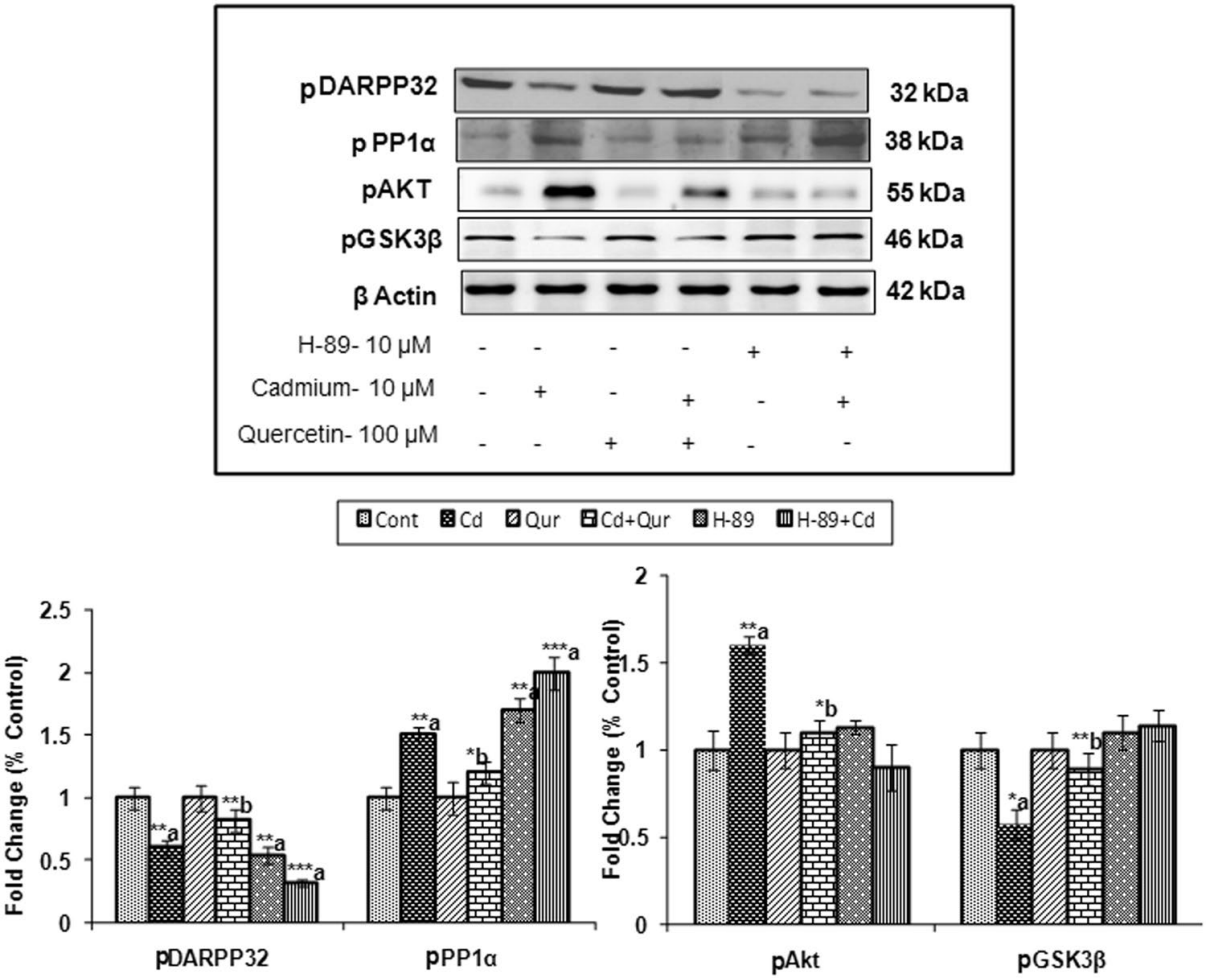
Effect of PKA inhibitor on dopamine receptor-mediated down streaming signaling during exposure to cadmium and quercetin. Quantitative analysis revealed that PC12 cells were preexposed to H-89 (Inhibitor of PKA) followed by the exposure to Cd (10 µM) and quercetin (100 µM) for 24 hr alters the postsynaptic signaling targets - DARPP-32 & PP1α with no significant change in the Akt and GSK-3β. β-actin was used as an internal control to normalize the data. Relative protein levels were quantified using Alpha Ease FC Stand Alone V.4.0 software after normalization with β-actin. The cropped gels are displayed for the clear representation. The data represent mean ± SE of three independent experiments; *p < 0.05; **p < 0.01, **p < 0.001; a-compared to control group; b-compared to cadmium exposed group; CONT – Control, Cd – Cadmium, QUR – Quercetin.

**Figure 11:**
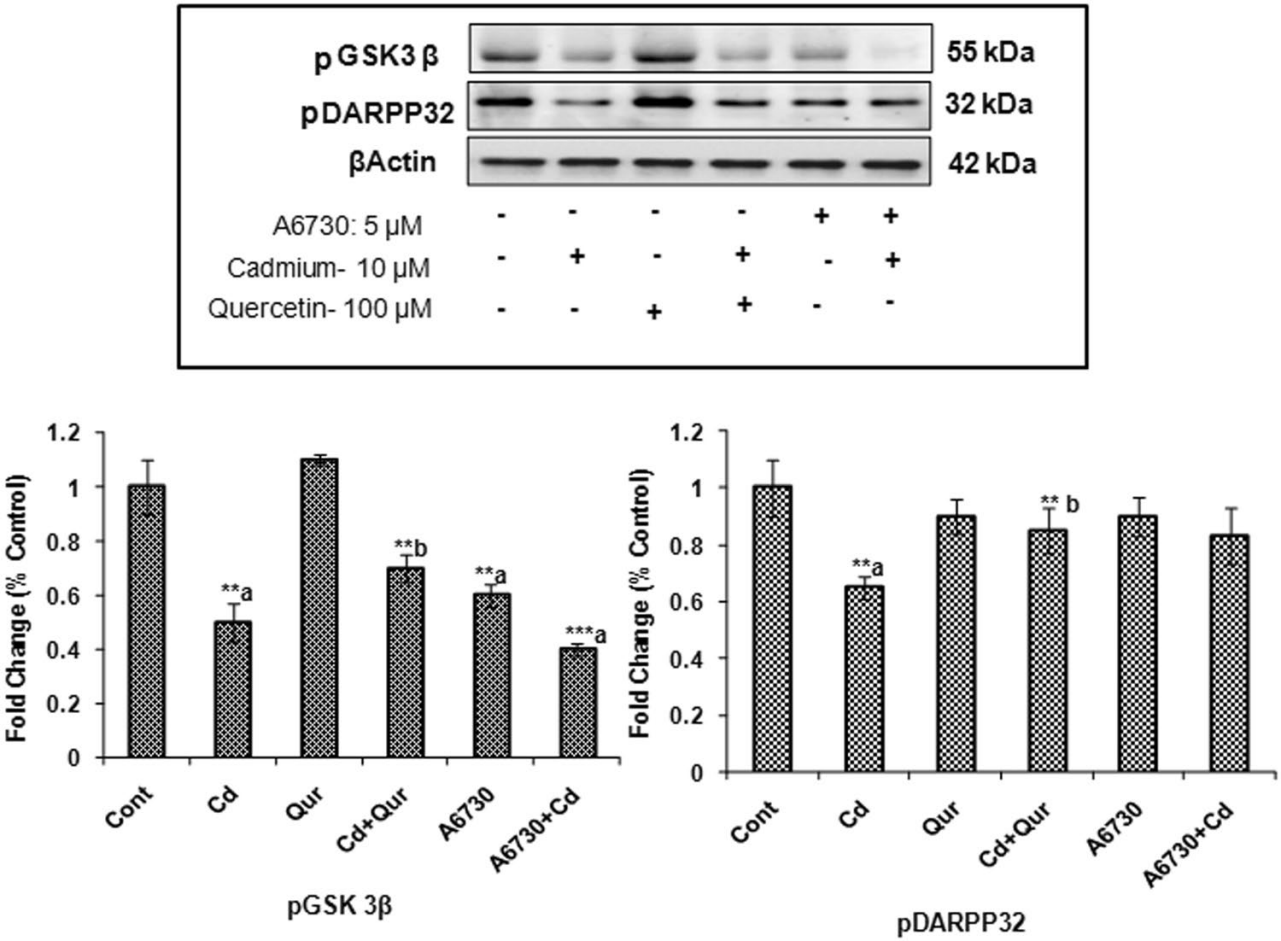
Effect of Akt inhibitor on dopamine receptor-mediated downstream signaling during exposure to cadmium and quercetin. Quantitative analysis revealed that PC12 cells were preexposed to A6730 (Inhibitor of Akt) followed by exposure to Cd (10 µM) and quercetin (100 µM) for 24 hr resulted in altering the postsynaptic signaling targets GSK-3β with no significant change in DARPP-32 & PP1α. β- actin was used as an internal control to normalize the data. Relative protein levels were quantified using Alpha Ease FC Stand Alone V.4.0 software after normalization with β-actin. The cropped gels are displayed for the clear representation. The data represent mean ± SE of three independent experiments; *p < 0.05; **p < 0.01, **p < 0.001.; a-compared to control group; b-compared to cadmium exposed group; CONT – Control, Cd – Cadmium, QUR – Quercetin.

Further, the involvement of dopamine receptors and its downstream signaling on exposure to cadmium and quercetin was confirmed using specific pharmacological inhibitors of PKA (H-89) and Akt (A6730). PC12 cells were initially treated with specific inhibitors of PKA or Akt for 1 hr before being exposed to cadmium (10 µM) and quercetin (100 µM) for 24 hr. Cadmium-induced changes were more pronounced in cells which were pre-treated with specific pharmacological inhibitors of PKA and Akt as compared to PC12 cells treated with cadmium alone. It was interesting that in the presence of a specific inhibitor of PKA, levels of DARPP-32 and PP1α were significantly altered while Akt and GSK-3β remained unchanged suggesting PKA independent mechanism. In the presence of specific inhibitor of Akt, there was a significant change in the levels of GSK-3β. However, no such change in the DARPP-32 was observed as compared to PC12 cells exposed to cadmium alone (Figs 10 and 11).

### *In Silico* studies- Modeling and Molecular Docking

#### Homology modeling studies

**Figure 12:**
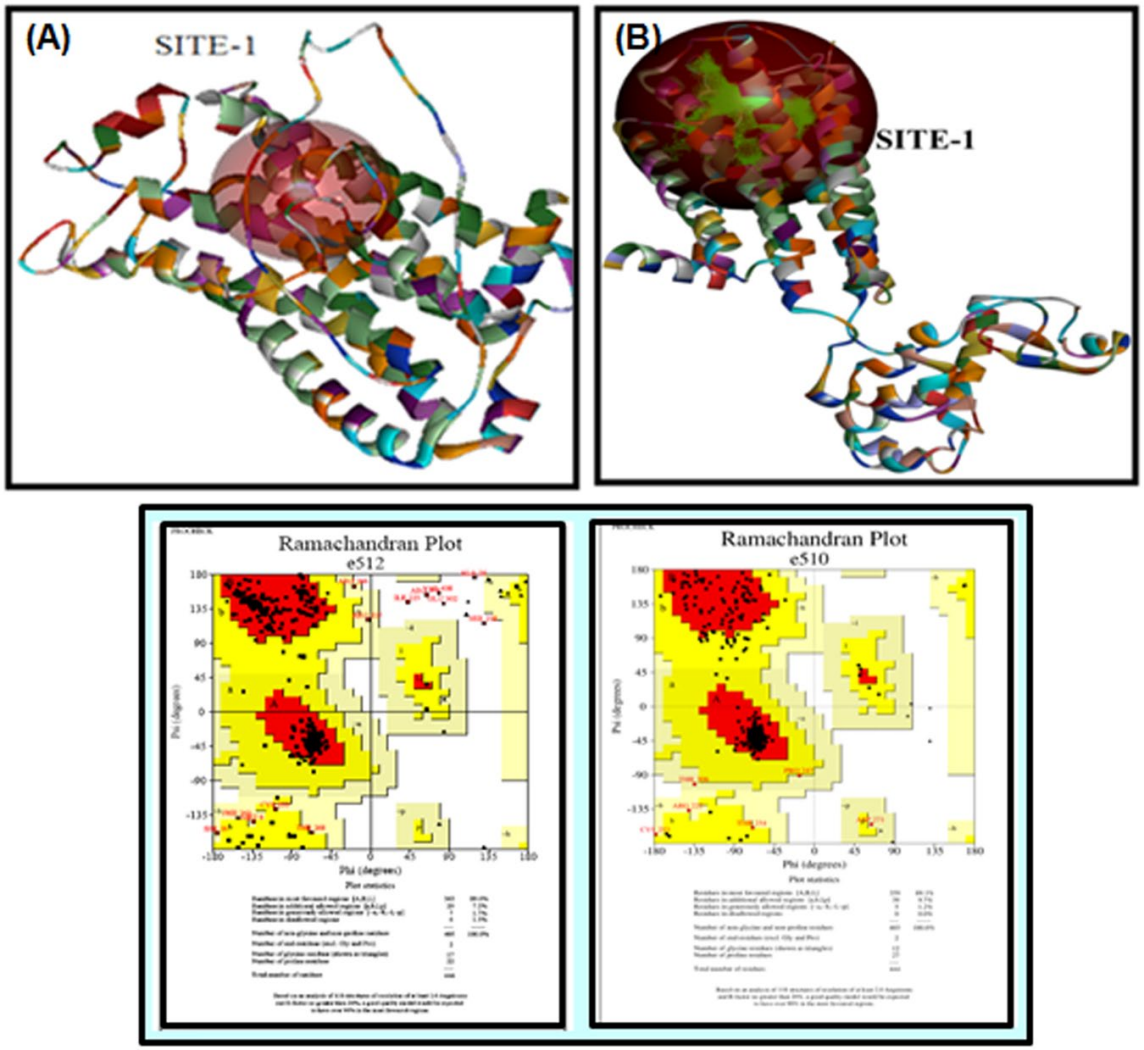
Homology modeling of DA-D1, DA-D2 Receptor. Modelling was done using the modeler 9.15. The representative images and Ramachandran Plot of DA-D1 (**A**) and DA-D2 (**B**) receptors has been shown.

3D-structure of the DA-D2 receptor for UniProt entry P61169 was modeled using the structure of the human DA-D3 receptor having PDB ID – 3PBL. It had 48% identity, 59% similarity and 95% query coverage with the DA-D2 receptor for *Rattus norvegicus*. The Ramachandran plot analysis of best model indicated 89.1% residues in favored region, 10.9% residues in allowed region and 0.0% residues in disallowed region. For DA-D1 receptors of *Rattus norvegicus* having UniProt ID – P18901, a homology model was constructed using template 3KJ6 (Methylated Beta2 Adrenergic Receptor) having 36% identity and 57% similarity. The Ramachandran plot analysis of the best model indicated 89.6% residues in the favored region, 8.9% residues in allowed region and 1.5% residues in the disallowed region (Fig. 12).

#### Docking and interaction analysis

**Figure 13:**
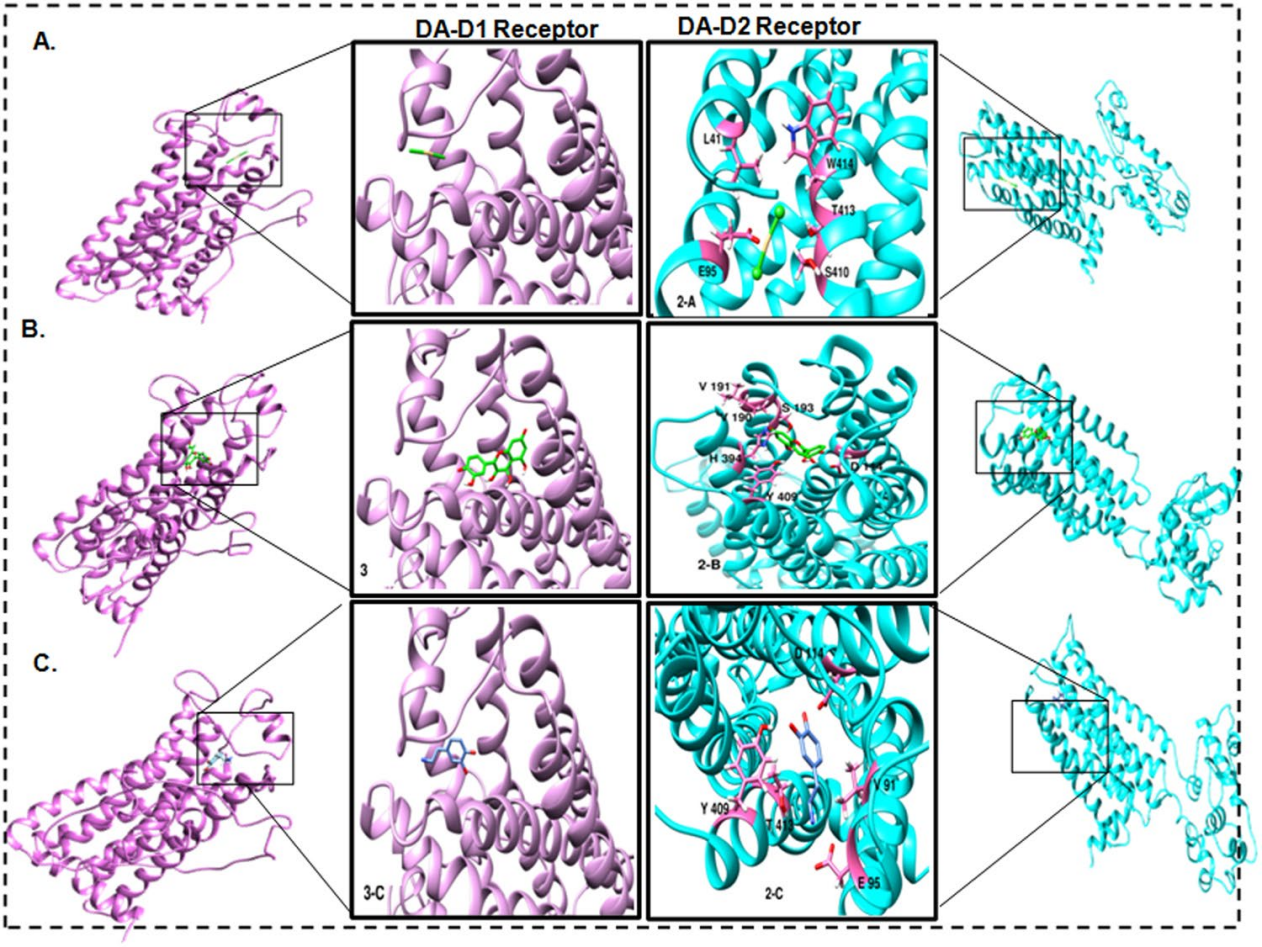
Ligand-protein interaction diagram (Ribbon view model) of DA-D1 and DA-D2 receptor of *Rattus norvegicus*. The residues interacting with their bound partners are indicated in pink color in atom view using Chimera. The docking of DA-D1 and DA-D2 receptors with CdCl_2_ (**A**), Quercetin (**B**) and Dopamine (**C**) molecules have been shown respectively. The docking was carried out involving CDOCKER.

Top 3 binding sites were used for docking all 3 compounds using CDOCKER module of Discovery Studio version 4.1. Lowest energy pose indicated CDOCKER energy of -61.4419, -46.6889 and -37.419 KJ/mol for compounds - CdCl_2_, quercetin, and dopamine respectively (Table 2). Non-bonded interaction module of Discovery Studio-4.1 identified residues namely L41, D95, S410, T413, W414 for compound CdCl_2_. For quercetin, residues identified were V111, D114, V190, S193, Y409, and H394. The dopamine, residues were V91, E95, D114, F409 and T413 (Fig. 13A–C). These non-bonded interactions include hydrogen bonding, hydrophobic interaction, electrostatic and Vanderwaal forces. After identification of a binding site for DA-D1 receptor model, it was docked with CdCl_2_, quercetin and dopamine molecules using CDOCKER module. Both for CdCl_2_ and quercetin, CDOCKER score was 23.5912 and 36.6108 respectively, which was quite low when compared to that of the DA-D2 receptor (Table 2) indicating that these molecules preferably interact with the later. For dopamine molecule, CDOCKER energy was 48.2473 KJ/mol and involves the interaction of residues namely Val91, Glu95, Asp114, Tyr409, Thr413.

**Table 2:** Summary of Interaction energies of cadmium, quercetin and dopamine ligand with DA-D1 and DA-D2 receptors.

S.No.	Receptor	Cadmium	Quercetin	Dopamine
1.	DA-D1 Receptor	−23.5912 K/Mol	−36.6108 K/Mol	−48.2473 K/Mol
2.	DA-D2 Receptor	−61.4419 K/Mol	−46.6889 K/Mol	−37.4190 K/Mol

Interactions energies were calculated using CDOCKER energies.

#### DFT Studies

**Figure 14:**
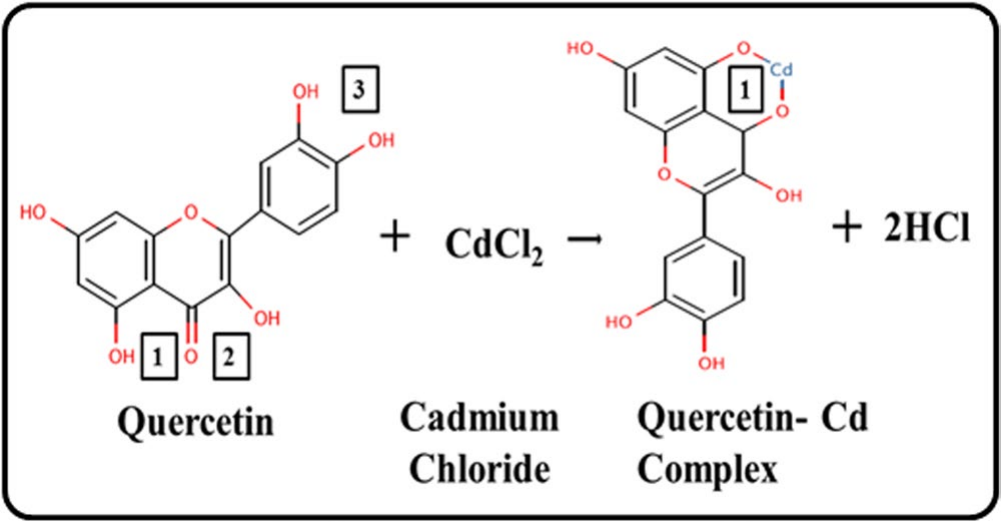
Proposed Quercetin–Cd complex formed by the Quercetin in the presence of CdCl_2_. The proposed complex has been suggested by the computational DFT studies.

The optimized stable structure of quercetin was solved using DFT method first. On the complete optimization of all four parameters (maximum force, maximum displacement, RMS force, RMS displacement) were converged. As previous studies suggested that cadmium ions interact with quercetin by interacting with its hydroxyl groups, DFT calculations were performed against all the possible sites where cadmium might interact with quercetin (Fig. 14). In case of site 2 and 3, the energies of the cadmium-quercetin complex were unable to converge to minima. While in case of Site 1, all the energies converged to minima, and the structure formed was optimized and found stable. The energy calculated was -1151.89985894 a.u. at this Site-1.

## Discussion

To the best of our knowledge, this is the first report that demonstrates targets associated with cadmium-induced dopaminergic alterations and evinces role of DA-D2 receptor-mediated postsynaptic signaling in regulating cadmium-induced motor dysfunctions. Further, the mechanism related to the ameliorative potential of quercetin, a polyphenolic flavonoid in cadmium-induced dopaminergic deficits and motor impairment has also been unraveled. Involvement of DA-D1 and DA-D2 type receptors in dopaminergic neurotransmission and in the integration of motor behavior and other pharmacological functions is largely accepted^13^. In the present study, cadmium treatment decreased mRNA and protein levels of DA-D2 receptors in the corpus striatum while there was no change both in mRNA and protein of DA-D1 receptors. Further, a decrease in the protein levels of DA-D2 receptors and no effect on DA-D1 receptors was also evident on cadmium exposure in PC12 cells *in vitro*. Interestingly, *in silico* studies carried out by us have complemented the findings indicating the selective effect of cadmium on DA-D2 receptors. Consistent with transcriptional and translational changes, a decrease in the binding of ^3^H-spiperone in the present study further confirms that cadmium affects the sensitivity of DA-D2 receptors in the corpus striatum. While brain dopamine receptors are an easy target of environmental chemicals^24–27^, selected changes either in DA-D1 or DA-D2 receptors have also been reported. Chronic exposure to arsenic at high dose (5 mg/L) in mice was found to decrease mRNA expression of DA-D2 receptors although there were no changes in DA-D1 receptors in striatum^24^.

Out of several factors associated with decreased binding of dopamine receptors, availability of dopamine at the synapse is an important regulator. The decrease in dopamine levels in the corpus striatum of developing rats prenatally exposed to cadmium has been reported^28,29^. Romero *et al*.^29^ also found decreased dopamine levels in the hypothalamus of cadmium-exposed rats while studying the protective effect of melatonin. In ongoing studies by us to assess the protective effect of quercetin in cadmium-induced alterations in brain biogenic amines, we also found that exposure to cadmium results in decreased dopamine levels in the corpus striatum. Further, *in silico* study provides interesting evidence that DA- D2 receptors are primarily affected by the cadmium. The decrease in DA-D2 receptors could be due to direct binding of cadmium at the competitive or non-competitive sites of dopamine on DA-D2 receptors.

The integrity of pre and postsynaptic signaling is important for dopaminergic neurotransmission. The role of tyrosine hydroxylase, a rate-limiting enzyme in dopamine synthesis and marker of dopaminergic neurons is well accepted^30^. The decrease in TH expression in the corpus striatum has been associated with reduced dopamine levels on exposure to a number of chemicals and drugs. Effect on DAT has been associated with decreased dopamine levels in clinical cases of Parkinson’s disease^31^. The decrease in the expression of TH and DAT in the corpus striatum on cadmium exposure in the present study may be correlated with reduced dopamine levels. Another important paradigm in dopaminergic signaling is VMAT2, a specific protein present in presynaptic terminals that transports cytoplasmic monoamine neurotransmitters including dopamine into synaptic vesicles. Although cadmium affected the expression of VMAT2, changes were not significant, which suggests that transport of dopamine into vesicles may not be affected. It is further interesting that similar changes were observed *in vitro* on exposure of PC12 cells to cadmium.

Exposure to cadmium in the present study resulted in a decrease of PKA and pDARPP-32 (Thr 34) phosphorylation with increased PP1α phosphorylation which led to alter CREB levels and affect motor behavior and motor coordination in rats. As there was no change in the expression of DA-D1 receptors in the corpus striatum on cadmium exposure, the PKA/DARPP-32/PP1α pathway appears to be modulated by DA-D2 receptors both *in vivo* and *in vitro*. During the course of signaling, it may affect the nuclear translocation of CREB, a transcription factor also known to be regulated by PKA. Inhibition of the expression of DARPP-32 was associated with a decrease in DA-D1 receptors in the corpus striatum of developing rats exposed to manganese^32^. Recently, a decrease in DA-D2 receptors on arsenic exposure in adult rats resulted in inhibiting expression of PKA and DARPP-32 and increasing the expression of PP1α although there was no change in the expression of DA-D1 receptors. The role of DARPP-32 and CREB in modulating motor activity and motor coordination is well reported^33^. Decreased motor activity in mice exposed to cannabinoids has been associated with impairment in PKA dependent phosphorylation of DARPP-32 (Thr34).

Besides canonical signaling, a multiple evidence exhibits involvement of β-arrestin-2/Akt/GSK-3β pathway in the regulation of dopamine-dependent behavior^15,25,34^. It has been found that both cAMP-dependent PKA/DARPP-32/PP1α and cAMP-independent β-arrestin/Akt/GSK-3β signaling could take place simultaneously in dopaminergic neurons and contribute equally in regulating motor functions. Pharmacological activation of Akt or inhibition of GSK-3β results in a reduction of dopamine associated locomotor function in DAT-KO mice and WT mice treated with amphetamine^35^. Further inhibition of GSK-3β inhibitors can reduce locomotor hyperactivity^25^. Conversely, transgenic GSK-3β mutant mice showed locomotor hyperactivity phenotype that is reminiscent of DAT-KO mice. Akt1-KO mice support the involvement of Akt inhibition in DA-D2 receptor-mediated behavioral response^36^. In the present study, increased phosphorylation of Akt (ser 473) and decreased GSK-3β (ser 9) in the corpus striatum exhibit involvement of Akt/GSK-3β pathway in cadmium-induced motor dysfunctions. It further suggests that decrease in DA-D2 receptors on cadmium treatment not only affects the phosphorylation of PKA but also affect phosphorylation of Akt and GSK-3β. Further, pharmacological studies involving specific inhibitors of PKA and Akt *in vitro* revealed that pretreatment of PC12 cells with PKA inhibitor affected the phosphorylation of DARPP-32 and PP1α while there was no change in the levels of Akt and GSK-3β. Similarly, inhibition of Akt in PC12 cells affected the phosphorylation of GSK-3β but not affected the phosphorylation of DARPP-32 and PP1α. It clearly indicates that both pathways are independent as inhibition of one pathway did not affect the integrity of other. These findings are interesting and suggest that besides canonical signaling mediated by cAMP-dependent PKA/DARPP-32/PP1α pathway, cadmium may also affect the Akt/GSK-3β signaling pathway and cause motor dysfunctions as evident by the decrease in distance traveled due to alterations in DA-D2 receptors in the corpus striatum. Ultrastructural changes in the present study exhibit synaptic loss in the neuropil region of the corpus striatum and thus indicates that cadmium affects the neuronal integrity. Apart from the loss of myelin sheath and vacuolization, mitochondrial swelling associated with damaged cristae indicate loss of permeability and cellular architecture. A significant decrease in Nissl’s positive neurons in the corpus striatum further suggests that cadmium may cause neuronal degeneration.

**Figure 15:**
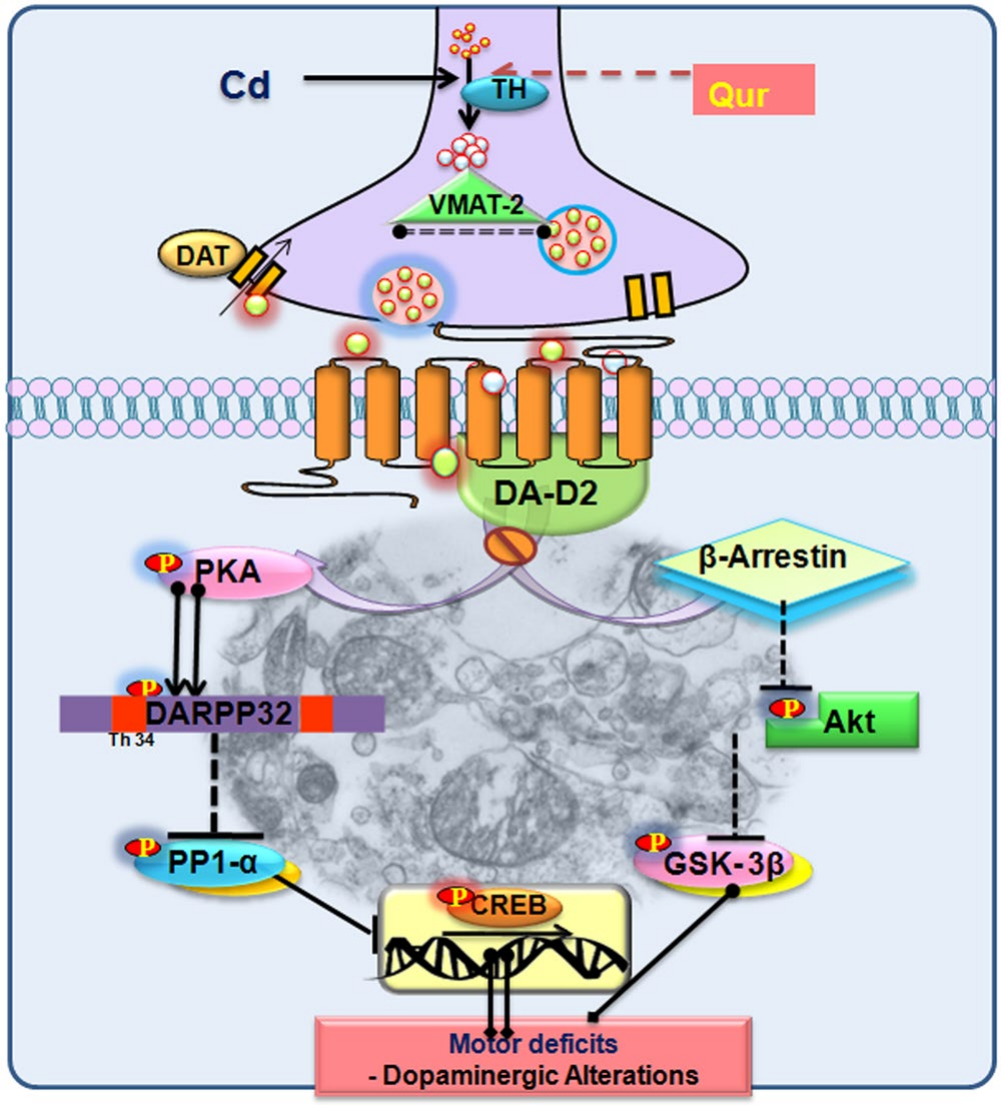
Schematic diagram illustrating the mechanism and target associated with cadmium-induced dopaminergic alterations and protective potential of quercetin in ameliorating such changes. The figure was designed and drawn by Richa Gupta and has no copyright material in it and is not submitted anywhere before for the publication. The figure shows the schematic representation of mechanism of cadmium-induced alteration in dopaminergic functions and associated motor alterations.

Given the ameliorative effect of quercetin in experimental models of Parkinson’s and Huntington’s diseases^22,37^, its protective potential was investigated in cadmium-induced dopaminergic dysfunctions. Combined treatment with quercetin and fish oil was found to protect depletion of dopamine levels in the striatum of rats chronically exposed to rotenone^23^. El-Horany *et al*.^22^ found that attenuation of rotenone-induced neurotoxicity in a rat model of Parkinson’s disease on quercetin treatment was due to an augmentation of autophagy associated with decreased oxidative stress and apoptosis. Neuronal death associated with enhanced astrogliosis and motor deficits in rats treated with 3-nitropropionic acid were also restored on supplementation with quercetin^37^. Being lipophilic due to multiple methylation of hydroxyl groups, quercetin may penetrate into the brain and modulate pharmacological functions^38^. The protective changes of quercetin were attributed to its antioxidant potential and capability to preserve mitochondrial integrity in rotenone-treated rats^22^. In the present study, simultaneous treatment with quercetin resulted to attenuate cadmium-induced decrease in DA-D2 receptors both in rat corpus striatum and PC12 cells. Further, cadmium-induced alterations in the expression of TH, DAT and DA-D2 receptor-mediated PKA signaling in the corpus striatum were also alleviated on simultaneous exposure with quercetin both *in vivo* and *in vitro*. The reason behind such modulation by quercetin may be due to its potent antioxidant and metal chelating properties. More interestingly, DFT studies carried out by us suggest that quercetin tends to form a complex with cadmium. It could, therefore, be attributed to the metal chelating property of quercetin and may have reduced free cadmium on simultaneous treatment. The results provide interesting evidence that motor deficits on cadmium exposure are associated with alteration in DA-D2 receptors involving both canonical cAMP-dependent PKA/DARRP-32/PP1α and non-canonical βarrestin/AKT/GSK-3β signaling in the corpus striatum. The results also reveal that quercetin has the potential to protect cadmium-induced dopaminergic dysfunctions (Fig. 15).

## Materials and Methods

### Chemicals and reagents

Cadmium chloride, quercetin, protease inhibitor and antibody for anti-mouse TH were purchased from Sigma Aldrich, India. The antibodies for anti-rabbit Dopamine transporter (DAT), anti-rabbit vesicular monoamine transporter (VMAT-2), anti-rabbit protein kinase A (PKA), anti-rabbit DARPP-32, anti-rabbit PP1α, anti-rabbit CREB, anti-rabbit Akt, and anti-rabbit β- actin were purchased from Cell Signaling Technology, USA. Secondary antibodies for goat anti-rabbit IgG HRP, goat anti-mouse IgG-HRP were also purchased from Cell Signaling Technology, USA. Other chemicals used in the study were of analytical grade and procured from local commercial sources.

### *In vivo* Studies-

#### Experimental animals and housing conditions

Adult male rats (180 ± 20 g) of Wistar strain obtained from the central animal breeding colony of CSIR-Indian Institute of Toxicology Research (CSIR-IITR), Lucknow, were housed in a room set with controlled temperature (25 ± 2 °C) with a 12-h light/dark cycle under standard hygiene conditions. The animals had free access to pellet diet procured from the national supplier and water *ad libitum*. The animals were acclimatized for 7 days before starting the experiment. The study was approved by the institutional animal ethics committee of CSIR-IITR, Lucknow (IITR/IAEC/50/13) and all experiments were carried out in accordance with the guidelines approved by the Committee for the Purpose of Control and Supervision of Experiments on Animals (CPCSEA), Ministry of Environment and Forests (Government of India), New Delhi, India.

#### Treatment procedure and sample preparation

Rats were randomly divided into four treatment groups. Rats in group I were treated with cadmium as cadmium chloride dissolved in distilled water (5 mg/kg body weight, p.o., once daily for 28 days). In Group II, rats received quercetin suspended in 0.1% tween 20 (25 mg/kg body weight, p.o., once daily for 28 days). Rats in group III were treated with cadmium and quercetin as in groups I and II respectively. Rats in group IV received distilled water and served as controls.

24 hours after the last dose of treatment, a separate set of rats was used for behavioral studies. For neurochemical studies, rats were decapitated, and their brains were taken out quickly and washed in ice-cold saline. The corpus striatum was dissected out following the standard procedure as described by Glowinski and Iversen^39^. For qRT-PCR studies, the corpus striatum was immersed in the Trizol reagent. For histological and ultrastructural studies, a set of rats was perfused with formaldehyde.

### Neurochemical Studies

#### Quantitative Real-Time PCR analysis

Quantitative Real-Time PCR (qRTPCR) was carried out to assess the transcriptional changes in DA-D1 and DA-D2 receptor type in the corpus striatum following the method as described earlier by Singh *et al*.^40^. Total RNA was isolated using the Trizol reagent (Life Technologies, USA). cDNA generation and RT-PCR was performed using High-Capacity cDNA Reverse Transcription Kit (Applied Biosystems, USA) and 1× Taq Man Universal PCR Master Mix (Applied Biosystems) was used. All PCR reactions were performed using standard PCR conditions. Relative expression was calculated using ΔΔCt values.

#### Assay of DA –D2 receptors using neurotransmitter receptor binding assay

Assay of DA –D2 receptors in the corpus striatum was carried out by radioligand receptor binding following the standard procedure as described earlier by Khanna *et al*.^41^. For preparing a crude synaptic membrane, the corpus striatum was homogenized in19 volumes of Tris-HCl buffer (5 mM, pH 7.4) and the homogenate was centrifuged (40,000 × g, 15 min, 4 °C). The supernatant thus obtained was discarded and the pellet suspended in homogenizing buffer (5 mM Tris-HCl, pH 7.4). The suspension was recentrifuged (40,000 × g, 15 min, 4 °C) and the pellet was finally suspended in Tris-HCl buffer (40 mM, pH 7.4) and stored at −80 °C.

The reaction mixture in a final volume of 1 ml contained a Tris-HCl buffer (40 mM, pH 7.4) containing ^3^H-Spiperone (18.5 Ci/mmol, 1 × 10^−9^ M). Binding incubations were carried out in triplicate for 15 min at 37 °C. A set of tubes containing haloperidol (1 × 10^−6^ M) as a competitor was also run simultaneously to determine the non-specific binding. Contents of the binding tubes were rapidly filtered on glass fiber discs (25 mm diameter, 1.0 μm pore size, Whatman GF/B) over vacuum manifold (Millipore, USA). The filter discs were rapidly washed with cold Tris-HCl buffer (40 mM, pH 7.4) twice in an attempt to remove unbound radioligand. The filter discs were transferred into vials and left overnight for drying. Scintillation mixture containing PPO, POPOP, naphthalene, toluene, and methanol (5 ml) were added to the vials, and bound radioactivity was counted by a β-scintillation counter (Packard, USA) at an efficiency of 30–40% for ^3^H. The nonspecific binding (in the presence of haloperidol) was subtracted from the total binding, and specific binding has been expressed as pmoles ^3^H-Spiperone bound/g protein. To further assess whether a decrease in the binding is due to alteration in the affinity (Kd) or the number of receptor binding sites (Bmax), Scatchard analysis was carried out using different concentrations of ^3^H-Spiperone (0.1–10 × 10^−9^ M).

#### Western blotting

To assess the expression of selected proteins, western blotting was carried out following the method as described earlier by Jamal *et al*.^42^. In short, an equal amount of lysate was separated using SDS-PAGE and transferred on to the PVDF membrane followed by blocking with blocking buffer (5% BSA). The membrane was incubated overnight at 4 °C with primary antibodies [TH (1:1000), DAT (1:1000), VMAT-2 (1: 1000), DA-D1 and DA-D2 receptor (1: 1000), PKA (1: 1000), pPKA (1: 1000), pDARPP-32 (1: 1000), pPP1α (1:1000), Akt (1:1000), pAkt (1:1000), GSK-3β (1:1000), pGSK-3β (1:1000), CREB (1:1000), pCREB (1:1000) and β- actin (1:1000). After washing, the membrane was incubated for 2 hrs at room temperature with secondary antibodies (1:2000). Blots were developed using Image Quant LAS500 (GE Healthcare Life sciences). β-actin was used as a loading control. Densitometric analysis was done to measure protein density and quantified using Alpha Ease FC Stand Alone V.4.0 software.

#### Protein estimation

Protein content was measured by the method of Lowry *et al*.^43^ using bovine serum albumin as a reference standard. A curve using varying concentrations of bovine serum albumin was constructed to calculate the concentration of protein in samples.

#### Neuronal degeneration- Histological studies

Coronal sections from the perfused brain were cut on a microtome (Microm HM 520, Labcon, Germany). The sections were stained with cresyl violet (1%) at 50 °C for 20 min  following dehydration and rehydration through graded series of alcohol following the standard procedure of Gupta *et al*.^44^. The sections containing Nissl positive neurons were imaged under light microscopy. The photomicrographs were processed on computerized software that enabled to assess the percent area of a selected field occupied by Nissl stained area. The quantification was performed using the image analysis system (Leica Qwin 500 image analysis software).

#### Transmission electron microscopy

Transmission electron microscopy (TEM) was performed in the corpus striatum dissected out from the brain of rats from each treatment group. Briefly, rats were anesthetized and perfused transcardially with paraformaldehyde (4%), glutaraldehyde (0.1%). Primary fixation was carried out for 2 h in glutaraldehyde (2.5%) followed by post-fixation in osmium tetroxide (1%) for 1–2 h at room temperature. The sample was dehydrated by graded series of acetone and embedded in Araldite and DDSA medium at 65 °C for 48 h. Further, the sample was cut into thin sections (60–90 nm) using ultramicrotome (Leica EM UC 67) and stained with uranyl acetate and lead citrate (2%) and examined over TEM (FEI, Tecnai G2 spirit model equipped with Gatan CCD/Orius camera at 80 KV).

### Neurobehavioral Studies

#### Spontaneous motor activity

Computerized Actimot (TSE, Germany) was used to assess the motor activity following the procedure as described by Yadav *et al*.^27^. The activity monitor is equipped with IR sensors and operates on light beam principle. Briefly, rats were placed at the center of the open field arena (45 cm × 45 cm) individually and allowed to move freely for 5 min. The parameters - total distance traveled, resting time, time moving, stereotypic counts and rearing were recorded automatically. Further, tracking pattern was recorded while monitoring the open field activity using computerized ACTIMOT equipped with software ActiMot2. The tracking pattern reflects the movement of animals.

#### Rota-rod performance

Motor coordination was assessed by Rotamex (Columbus, USA) following the method as described by Yadav *et al*.^27^. Initially, rats received training on the rotating rod of Rotamex at a constant speed of 8 rpm. During the final trial, the rotational speed increased gradually from 4 to 40 rpm over 300 s. The latency to fall from the rotating rod was measured, and scoring was carried out by a person unaware of the treatment status. Time spent on the rotating rod was the basic criterion to assess the effect on motor coordination.

#### Grip strength

Computerized grip strength meter (TSE, Germany) was used to assess the forelimb grip strength in rats and procedure as described by Shukla *et al*.^45^ was followed. Rats were carefully held from the nape and base of the tail and forelimbs were placed on the tension bar. Rats were pulled back gently until they released the bar. The reading was recorded automatically on the computer. Five successive pulls were tried for each animal by a person unaware of their treatment status. The mean of all values was taken and processed for statistical analysis. Results are expressed in Pounds.

### *In vitro* Studies

#### Cell culture and NGF induced neuronal differentiation

PC12, a cell line derived from the pheochromocytoma of the rat adrenal medulla, originally procured from National Centre for Cell Science (NCCS), Pune has been maintained at *In vitro* Toxicology Laboratory at CSIR-IITR, Lucknow. In brief, cells were cultured in standard conditions in RPMI cell culture medium supplemented with FBS (5%), HS (10%), sodium bicarbonate (0.2%) and antibiotic/antimycotic cocktail (1%) under CO2 (5%), and high atmospheric humidity at 37 °C. For all studies, cells at passage 6–12 were used. The viability of cells was measured by trypan blue dye exclusion and batches of cells having more than 95% viability were used in the study. After passage # 6, the cells were plated on poly-L Lysine (PLL) coated flasks. For inducing neuronal differentiation, cells were incubated in medium containing NGF (100 ng/ml). For experimental purpose, confluent growing cells were sub-cultured in PLL pre-coated six-well culture plates and 75-Cm^2^ culture flasks.

#### MTT Assay

Cell viability was ascertained by MTT assay as described by Agarwal *et al*.^46^. In brief, cells (1 × 10^4^ cells/ml) were seeded in 96-well plates for 24 h under a high humid environment with CO2 (5%) and atmospheric air (95%) at 37 °C. The medium was aspirated, and cells were exposed to variable concentrations of cadmium (0.1–1000 µM), quercetin (0.1–1000 µM) for 24–96 hr. Tetrazolium bromide salt (10 µl/well; 5 mg/ml of stock in PBS) was added 4 h prior to the completion of incubation in respective cases. Plates were incubated at 37 °C for 4 h, MTT solution removed and the cells were lysed using a culture grade DMSO by pipetting up and down several times until the content was homogenized. The color was read at 550 nm using multi-well microplate reader (Synergy HT, Bio-Tek, USA).

#### Western blotting (*In vitro*)

Western blotting was conducted following the protocol as described earlier by Kumar *et al*.^47^. After respective exposures with cadmium or quercetin alone or in combination, cells were scraped, pelleted, and lysed using CelLytic M Cell Lysis Reagent (Sigma) in the presence of protein inhibitor cocktail. An equal amount (40 μg/well) of denatured protein (determined by Bradford method) was loaded on SDS-PAGE, blotted onto PVDF by the wet transfer method. Nonspecific binding was blocked with BSA (5%) for 1 h. After blocking, the membrane was incubated overnight at 4 °C with primary antibodies specific for TH (1;1000), DAT (1:1000), DA-D1 and DA-D2 receptor (1: 1000), PKA (1:000), phospho-PKA (1:1000), Akt (1:1000), phospho-Akt (1:1000), phospho-DARPP-32 (1: 1000), phospho-PP1α (1:1000), GSK-3β (1:1000), phospho-GSK-3β (1:1000) and β-actin (1;2000) in blocking buffer (pH 7.5). The membrane was incubated for 2 h at room temperature with HRP conjugated secondary antibodies (corresponding to anti-primary immunoglobulin G). The blots were developed using Super Signal West Femto Chemiluminescent Substrate (ThermoFisher Scientific). The densitometry for protein-specific bands was conducted in Gel Documentation System (Alpha Innotech) with the help of Alpha Ease FC Stand Alone V.4.0 software. The marker proteins analyzed to study the altered expression were same as studied in the *in vivo* studies.

#### Studies with pharmacological inhibitors

Role of DA-D2 and its downstream signaling pathway was confirmed using specific pharmacological inhibitors. The cells were seeded in PLL pre-coated 96 well culture plates and allowed to adhere for 24 h prior to the experimental exposure. Prior to exposure to cadmium (10 μM) and quercetin (100 μM) for 24 h, cells were exposed to pharmacological inhibitors of Akt (A6730: 5 μM), PKA (H-89: 10 μM) for 1 h respectively.

### *In silico* Studies

To assess the molecular level interaction of cadmium chloride, quercetin with DA-D1 and DA-D2 receptors, computational studies were carried out involving homology modeling and molecular docking.

### Homology modeling of DA-D1 and DA-D2 receptors

The protein sequence of the DA-D1 and DA-D2 receptor in animal model *Rattus norvegicus* was retrieved from UniProt database (P18901, P61169). Homology-based model for DA –D1 and DA- D2 receptor was built by homology modeling approach using Modeller version 9.15^48^. Templates for modeling protein sequence were identified using blastP tool against Protein Data Bank^49^. Homolog having good structural similarity was used to build 200 protein models, and top 20 models selected by their lowest DOPE score were analyzed for structural stability using RAMACHANDRAN PLOT analysis feature of PROCHECK server^50^.

### Ligand preparation

Structures of CdCl_2_, quercetin, and dopamine were built and minimized using Marvin Sketch version 6.1.2 from ChemAxon and then clean in 3D using Steepest Descent method.

### Molecular docking

Ligand binding pockets on DA-D1 and DA- D2 protein were identified using binding site identification module of Discovery Studio 4. Top 3 sites identified in D2 receptor were docked with all 3 compounds (Quercetin, Dopamine and CdCl_2_) using CDOCKER module of DS version 4.1^51^. UCSF Chimera was utilized for image generation^52^.

### DFT Studies

For predicting the structure of Cd-Quercetin complex, DFT calculations were performed with the Gaussian09 package^53^. DFT studies were carried out using Ground State, Default Spin and RB3LYP method. The basic set used was LanL2DZ. Guess method used was Default, and solvation was performed without any constraint using the SMD model in water. At 298 K and 1 atm Pressure, frequency analysis was carried out to confirm that each structure is a local minimum with no imaginary frequency or a transition state with only one imaginary frequency. The 3D images of the calculated structure were prepared using Gauss-View 5.0^54^.

### Statistical analysis

Data has been expressed as mean ± SEM. To compare the significance of all pairs of columns, Newman–Keuls method using one-way analysis of variance (ANOVA) was carried out. GraphPad prism3 software was used. F values represented in the results exhibit the variability between, and within the groups and reflect the degree of freedom (df). p values represent the probability. Interestingly, these two factors are dependent on each other. Further, p-value up to 0.05 has been considered significant.

## Electronic supplementary material

Supplementary file
