# Supplementary material for: Involvement of PKA/DARPP-32/PP1α and β- arrestin/Akt/GSK-3β Signaling in Cadmium-Induced DA-D2 Receptor-Mediated Motor Dysfunctions: Protective Role of Quercetin
Source: Sci Rep. 2018 Feb 6;8:2528. doi: 10.1038/s41598-018-20342-z (PMC5802731; doi:10.1038/s41598-018-20342-z)

Involvement of PKA/DARPP32/PP1α and β- arrestin/Akt/Gsk-3β signaling in Cadmium Induced DA-D2 Receptor Mediated Motor dysfunctions: Protective Role of Quercetin

Richa Gupta[1, 2], Rajendra K. Shukla[1], Ankita Pandey[1], Tanuj Sharma[3], Yogesh Dhuriya[1], Pranay Srivastava[1], Manjul P. Singh[2], Mohammad Imran Siddiqi[3] , AB Pant[1] and Vinay K. Khanna[1]

[1]Developmental Toxicology Laboratory
Systems Toxicology and Health Risk Assessment Group,
CSIR-Indian Institute of Toxicology Research, Vishvigyan Bhawan,
31 Mahatma Gandhi Marg, Lucknow – 226 001,
Uttar Pradesh, India

[2]School of Pharmacy, Babu Banarsi Das University, Faizabad Road,
Lucknow – 226 028; Uttar Pradesh; India

[3]Computational Biology Bioinformatics & Chemoinformatics
CSIR-Central Drug Research Institute
Sitapur Road, Sector 10 Jankipuram Extension
Lucknow – 226 031, Uttar Pradesh, India

***Corresponding Author**

Dr. Vinay K. Khanna
Senior Principal Scientist
Developmental Toxicology Laboratory
System Toxicology and Health Risk Assessment Group
CSIR-Indian Institute of Toxicology Research
Lucknow – 226 001, UP (India)
E-mail:vkkhanna1@rediffmail.com

Supplementary File 1 - Neuronal Differentiation of Pc12 ells using NGF:

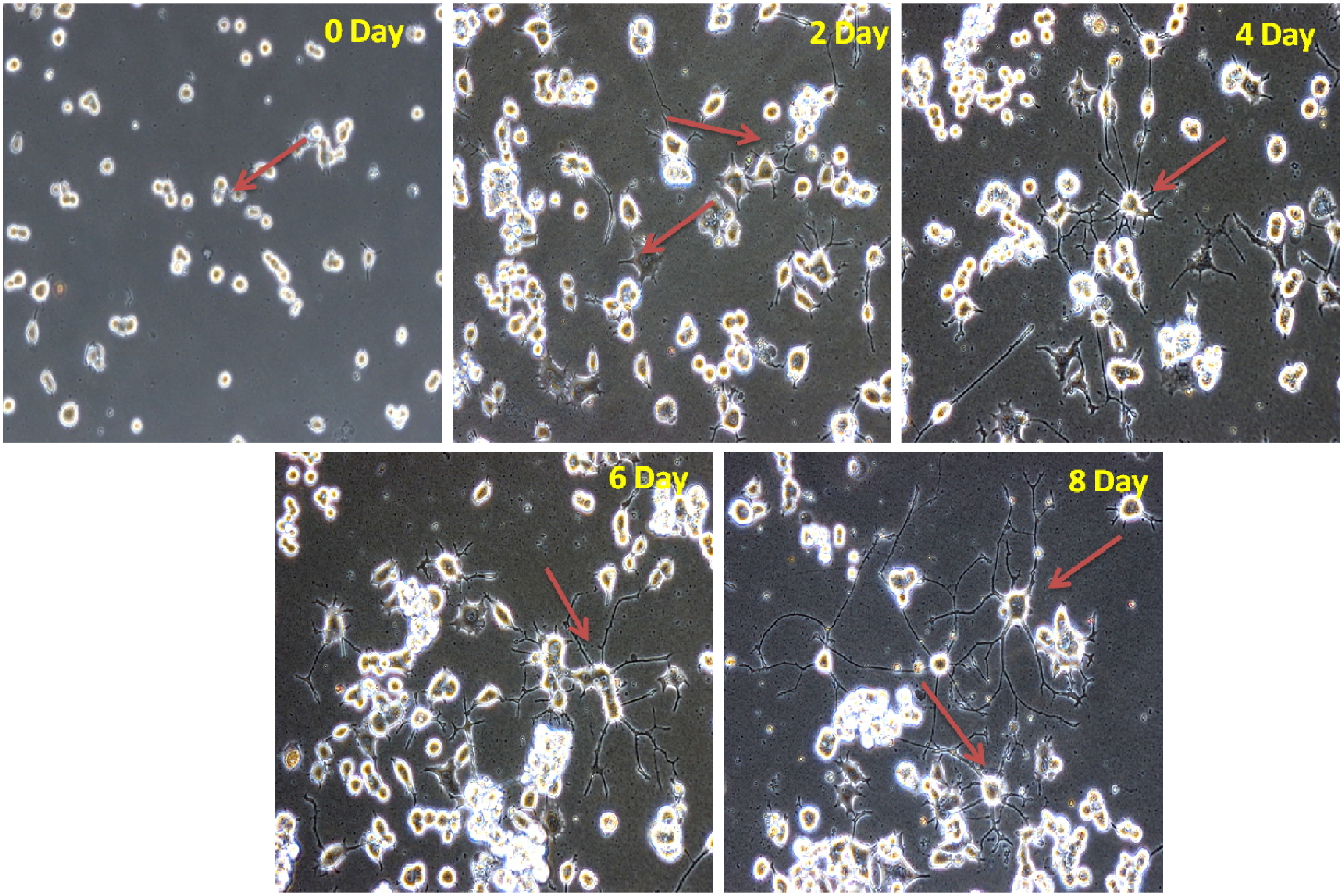

Supplementary File 2- Full Length blot of DARPP32 and GSK 3β

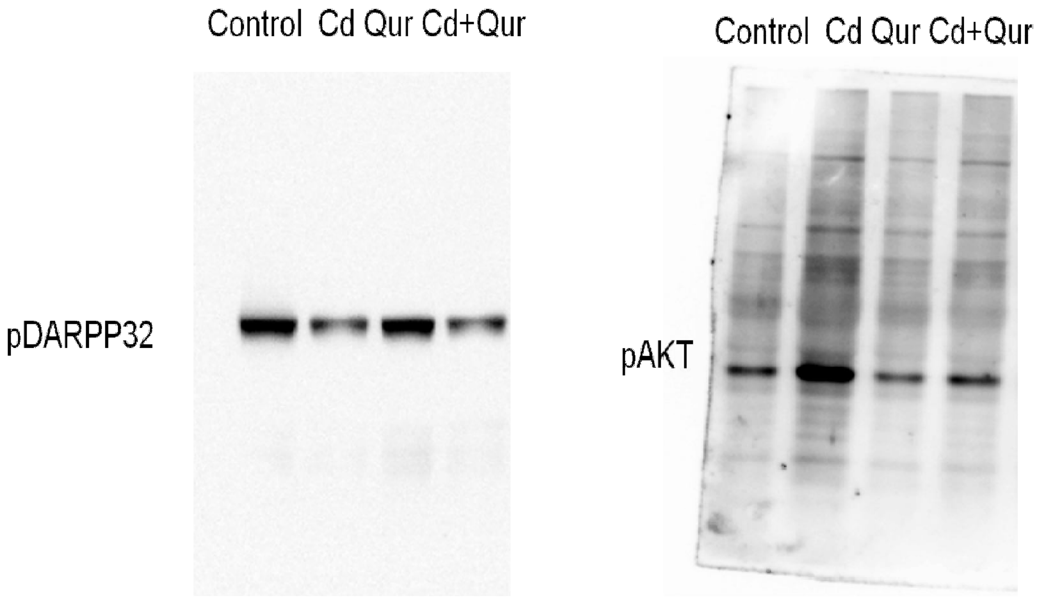

Supplement: Supplementary file 1 — Supplementary file [file 41598_2018_20342_MOESM1_ESM.pdf]
